# Functional investigation of the coronary artery disease gene *SVEP1*

**DOI:** 10.1007/s00395-020-00828-6

**Published:** 2020-11-13

**Authors:** Michael J. Winkler, Philipp Müller, Amin M. Sharifi, Jana Wobst, Hanna Winter, Michal Mokry, Lijiang Ma, Sander W. van der Laan, Shichao Pang, Benedikt Miritsch, Julia Hinterdobler, Julia Werner, Barbara Stiller, Ulrich Güldener, Tom R. Webb, Folkert W. Asselbergs, Johan L. M. Björkegren, Lars Maegdefessel, Heribert Schunkert, Hendrik B. Sager, Thorsten Kessler

**Affiliations:** 1 Department of Cardiology, German Heart Centre Munich, Technical University of Munich, Munich, Germany; 2 German Centre for Cardiovascular Research (DZHK e.V.), Partner Site Munich Heart Alliance, Munich, Germany; 3 Vascular Biology and Experimental Vascular Medicine Unit, Department of Vascular and Endovascular Surgery, Klinikum rechts der Isar, Technical University Munich, Munich, Germany; 4 Division of Heart and Lungs, Department of Cardiology, University Medical Center Utrecht, Utrecht, The Netherlands; 5 Department of Genetics and Genomic Sciences, Icahn Institute for Genomics and Multiscale Biology, Icahn School of Medicine at Mount Sinai, New York, NY USA; 6 Department of Cardiovascular Sciences, University of Leicester, and National Institute for Health Research (NIHR) Leicester Cardiovascular Biomedical Research Centre, Leicester, UK; 7 Institute of Cardiovascular Science, Faculty of Population Health Sciences, and Health Data Research UK and Institute of Health Informatics, University College London, London, UK; 8 Integrated Cardio Metabolic Centre, Department of Medicine, Karolinska Institutet, Karolinska Universitetssjukhuset, Huddinge, Sweden; 9 Department of Physiology, Institute of Biomedicine and Translational Medicine, University of Tartu, Tartu, Estonia

**Keywords:** SVEP1, Atherosclerosis, Coronary artery disease, Genetics

## Abstract

A missense variant of the sushi, von Willebrand factor type A, EGF and pentraxin domain containing protein 1 (SVEP1) is genome-wide significantly associated with coronary artery disease. The mechanisms how SVEP1 impacts atherosclerosis are not known. We found endothelial cells (EC) and vascular smooth muscle cells to represent the major cellular source of SVEP1 in plaques. Plaques were larger in atherosclerosis-prone Svep1 haploinsufficient (*ApoE*^−/−^*Svep1*^+/−^) compared to Svep1 wild-type mice *(ApoE*^−/−^*Svep1*^+/+^) and *ApoE*^−/−^*Svep1*^+/−^ mice displayed elevated plaque neutrophil, Ly6C^high^ monocyte, and macrophage numbers. We assessed how leukocytes accumulated more inside plaques in *ApoE*^−/−^*Svep1*^+/−^ mice and found enhanced leukocyte recruitment from blood into plaques. In vitro, we examined how SVEP1 deficiency promotes leukocyte recruitment and found elevated expression of the leukocyte attractant chemokine (C-X-C motif) ligand 1 (*CXCL1*) in EC after incubation with missense compared to wild-type SVEP1. Increasing wild-type SVEP1 levels silenced endothelial CXCL1 release. In line, plasma Cxcl1 levels were elevated in *ApoE*^−/−^*Svep1*^+/−^ mice. Our studies reveal an atheroprotective role of SVEP1. Deficiency of wild-type Svep1 increased endothelial *CXCL1* expression leading to enhanced recruitment of proinflammatory leukocytes from blood to plaque. Consequently, elevated vascular inflammation resulted in enhanced plaque progression in Svep1 deficiency.

**Electronic supplementary material:**

The online version of this article (10.1007/s00395-020-00828-6) contains supplementary material, which is available to authorized users.

## Introduction

Coronary artery disease (CAD) and myocardial infarction (MI) are the leading causes of death in industrialized countries [4]. Genome-wide association studies have identified more than 160 variants, mainly located in the non-coding genome, that are associated with CAD/MI [13]. In an exome-wide association study, a coding missense variant in *SVEP1* (rs111245230, p.D2702G) is genome-wide significantly associated with CAD/MI (Odds Ratio 1.14 per risk allele) [24]. *SVEP1* encodes sushi, von Willebrand factor type A, EGF and pentraxin domain containing protein 1 (SVEP1), an extracellular matrix (ECM) protein that binds to integrin α9β1 [32], which is expressed in lymphatic system endothelial cells (EC) [3]. Integrin α9β1 plays a role in adhesion and transendothelial migration of peripheral neutrophils, hence promoting inflammatory processes. In context of stroke, impairing the function of integrin α9β1 led to reduced thrombosis and inflammation and limited short- and long-term brain damage [10]. Svep1 and integrin β1-pathways are also involved in maintaining vascular integrity. This is achieved by induction of *Tie1* expression via binding of Svep1 to angiopoietin-2 [23]. Under acute inflammatory conditions, Tie1 is cleaved, resulting in reduced Tie1 quantity on the cell surface and subsequently weakening cell–cell junctions. Thus, reduced *Tie1* expression leads to vascular destabilization and Svep1 was found to be crucial to maintain vascular integrity under baseline conditions [19]. Integrin β1 pathways are also involved in cell migration to the lymphatic system. In line, absence of Svep1 has been found to impair lymphatic vessel formation in zebrafish [17] and mice [23].

Despite its strong association with CAD, the role of SVEP1 in atherosclerosis has yet to be determined. In this project, we sought to investigate the functional involvement of SVEP1 in mice and patients with atherosclerosis.

## Methods

### SVEP1 expression in the STARNET study

The Stockholm–Tartu Atherosclerosis Reverse Networks Engineering Task study (STARNET) subjects recruitment and tissue collection were described previously [14]. Briefly, patients with coronary artery disease (CAD) who were eligible for open-thorax surgery at the Department of Cardiac Surgery, Tartu University Hospital in Estonia as well as control subjects without CAD were enrolled after informed consent. Tissue biopsies were obtained to study tissue-specific gene expression and the disease. Tissues were rinsed and RNA was extracted as described previously [14]. In the case–control matched study, cases and controls with matched age, gender, and BMI were selected and sequenced for whole transcriptome. Samples were sequenced with poly(A)+selection on Illumina HiSeq with single-end at read lengths of 100 base pairs. Quality control was performed using FASTQC [1] checking raw sequence data for per-base quality, per-sequence quality, number of duplicate reads, number of reads with an adaptor, sequence length distribution, per-base GC content, per-sequence GC content and Kmer content. GENCODE was used as reference annotation to quantify gene and isoform expression. Sequencing reads (fastq files) were mapped with STAR [12] onto the human genome. Raw reads were summarized by feature counts [20]. Differential gene expression between cases and controls was analyzed using R package limma [22].

### Human carotid artery plaque specimen, immunohistochemistry, and single-cell RNA sequencing

Human carotid arterial plaque material of the Munich Vascular Biobank [29] was sampled during carotid endarterectomy (CEA), fixed for 48 h in 2% zinc–paraformaldehyde at room temperature, paraffin-embedded, and finally cut into 5-μm thick slides. Per carotid plaque specimen, four slides were stained with hematoxylin and eosin (HE) as well as Elastica van Gieson’s staining. For immunohistochemical analysis, consecutively cut tissue sections of 3 µm thickness were deparaffinized, permeabilized with H_2_O_2_ and blocked in milk powder. Sections were then incubated for 1 h at room temperature with primary antibodies against SVEP1 (HPA020610, Sigma-Aldrich, St. Louis, MO, USA), CD31 (M0814, DAKO, Carpinteria, CA, USA), CD68 (M0823, DAKO, Carpinteria, CA, USA), smooth muscle a-actin/SMA (M0635, DAKO, Carpinteria, CA, USA). SVEP1 staining was established and compared to isotype control IgG antibody/no primary control (Suppl. Fig. S1). Secondary antibodies were provided with the DAKO REAL Detection Kit Rabbit/Mouse (DAKO, Carpinteria, CA, USA). Detection was mediated by a 3,3′-diaminobenzidine-coupled reaction according to the manufacturer’s instructions. Nuclear staining with hematoxylin was performed before dehydrating the slides in an increasing ethanol row followed by xylene. Slides were finally mounted with EUKITT (Kindler, Bobingen, Germany) and imaged.

Single-cell RNA sequencing data from 18 (14 male and 4 female) individuals were obtained from Depuydt et al. [9] within the Athero-Express Biobank Study (www.atheroexpress.nl) [36], an ongoing biobank study at the University Medical Centre Utrecht (UMCU). Details are available in the Supplemental Material.

### Primary cells

Human coronary artery smooth muscle cells (CASMC), human aortic adventitial fibroblasts (AAF), human aortic endothelial cells (AEC), human aortic smooth muscle cells (ASMC), human coronary artery endothelial cells (CAEC), human coronary artery smooth muscle cells (CASMC), and human umbilical vein endothelial cells (HUVEC) were purchased from commercial suppliers (CASMSC: Cell Applications, San Diego, CA, USA; AAF, AEC, CAEC, CASMC: from ScienCell, Carlsbad, CA, USA; HUVEC: PromoCell, Heidelberg, Germany). Monocytes were isolated from whole blood from one healthy individual using a combined Ficoll-Paque density gradient and a CD14 magnetic bead separation approach. Details on isolating blood monocytes as well as the investigated primary cells are available in the Supplemental Material.

### Isolation of nucleic acids, cDNA synthesis, and (quantitative) polymerase chain reaction

RNA from isolated blood monocytes was extracted using the RNeasy Mini Kit (Qiagen, Hilden, Germany) according to the manufacturer’s recommendations. Otherwise, cells were washed with Dulbecco’s PBS (Biochrom, Berlin, Germany), lysed by addition of 500 µl TRIzol (Thermo Fisher Scientific, Waltham, MA, USA) and stored at − 80 °C. After thawing, 120 µl DEPC treated H_2_O (Invitrogen, Carlsbad, CA, USA) and 100 µl chloroform were added. After 10 min incubation at room temperature, the mixture was transferred to pre-chilled phase lock tubes (Quantabio, Beverly, MA, USA) and centrifuged at 13,000 rpm and 4 °C for 10 min. 280 µl of isopropanol and 1.5 µl of GlycoBlue (Invitrogen, Carlsbad, CA, USA) were added to the aqueous phase. The isolated RNA was incubated at − 20 °C for 30 min and pelleted by centrifugation at 14,000 rpm for 30 min at 4 °C and washed twice with 75% ethanol. After evaporation of ethanol, RNA pellet was resuspended in 14 µl of DEPC treated water. After DNA digestion with DNase I (Thermo Fisher Scientific, Waltham, MA, USA) according to the manufacturer’s recommendation, cDNA was synthesized by reverse transcription utilizing the Maxima H Minus Reverse Transcriptase kit (Thermo Fisher Scientific, Waltham, MA, USA). For endpoint polymerase chain reaction (PCR), 20 ng of cDNA were used in each reaction with Quick-Load® Taq 2 × Master Mix (New England Biolabs, Ipswich, MA, USA) and the following oligonucleotide primers: *SVEP1-for* 5′-GCA ACT TGG GCG TGG TTA TG-3′, *SVEP1-rev* 5′-CAC ACC GCT GAC CTG TGT AA-3′; *Svep1-for*: 5′-ACC ATA CAC GGG AGA TGG GA-3′; *Svep1-rev*: 5′-GGG TGT AGC CCT CTT TGC AT-3′; *RPLP0-for* 5′-GGC ACC ATT GAA ATC CTG AGT G-3′, *RPLP0-rev* 5′-GAT GAC CAG CCC AAA GGA GAA G-3′; *Rplp0-for*: 5′-TCA CTG TGC CAG CTC AGA AC-3′; *Rplp0-rev*: 5′-ATC AGC TGC ACA TCA CTC AGA-3′. Reactions were performed over 40 cycles (15 s at 95 °C, 30 s at 60 °C, 1 min at 68 °C). and loaded to 1% (w/v) agarose gels dyed with peqGREEN (VWR, Darmstadt, Germany).

Real-time quantitative PCR was performed using TaqMan Universal Master Mix II (Thermo Fisher Scientific, Waltham, MA, USA) in a total volume of 20 µl using MicroAmp Fast 96-Well 0.1 ml reaction plates (Thermo Fisher Scientific, Waltham, MA, USA). All reactions were conducted over 40 cycles and performed in duplicates on a ViiA 7 system using Taqman probes (both Thermo Fisher Scientific, Waltham, MA, USA). The Taqman probes which were used to detect genes of interest are listed in Suppl. Table S2. *GAPDH/Gapdh* or *RPLP0*/*Rplp0* were used as housekeeping genes. Expression of genes of interest comparing SVEP1 and SVEP1_p.D2702G was analyzed using the 2^−ΔΔ*Ct*^ method. Absolute mRNA levels were compared using 2^−Δ*Ct*^ values.

### Mouse studies

B6N(Cg)-*Svep1*^*tm1b(EUCOMM)Hmgu*^/J (reporter-tagged deletion allele, https://www.informatics.jax.org/allele/MGI:5509058; *Svep1*^+/−^), *Ubc-GFP* mice (C57BL/6-Tg(UBC-GFP)30Scha/J), and B6.129P2-*Apoe*^tm1Unc^/J (*ApoE*^−/−^) mice were purchased from the Jackson Laboratory (Bar Harbor, ME, USA). *Svep1*^+/−^ mice and *ApoE*^−/−^ mice were crossbred to generate *ApoE*^−/−^*Svep1*^+/−^ mice. For experiments, *ApoE*^−/−^*Svep1*^+/−^ mice were mated with *ApoE*^−/−^*Svep1*^+/+^ mice for at least four generations to receive *ApoE*^−/−^*Svep1*^+/−^ and *ApoE*^−/−^*Svep1*^+/+^ littermates in a 1:1 ratio. All animal experiments were approved by the local animal welfare committee (55.2-1-54-2532-49-2016). To study atherosclerotic plaque formation, 8 weeks old *ApoE*^−/−^*Svep1*^+/−^ and *ApoE*^−/−^*Svep1*^+/+^ mice of both genders were fed a high cholesterol diet (TD88137; Envigo, Huntingdon, United Kingdom) for 12 weeks. Weight measurements were performed at beginning and end as well as weekly during the diet. After 12 weeks of high cholesterol diet, mice were sacrificed. Blood was collected by cardiac puncture using a 50 mM ethylenediaminetetraacetic acid (EDTA) solution (St. Louis, MO, USA) as anticoagulant and used for measurements of total cholesterol using routine methods in the institutional clinical chemistry department as well as flow cytometry analyses of blood leukocyte counts. Plasma was also stored for subsequent analyses.

### Histology

The aortic root was embedded in Tissue-Tek O.C.T. Compound (Sakura Finetek, Alphen aan den Rijn, Netherlands) and snap-frozen in a methylbutane bath cooled with dry ice. Cryosections of 5 µm were obtained and used for hematoxylin–eosin (Carl Roth, Karlsruhe, Germany), Masson's trichrome (Sigma-Aldrich, St. Louis, MO, USA) as well as monocyte and macrophage (MOMA-2; ab33451, Abcam, Cambridge, UK) and myeloid cell (CD11b; 101,202, BioLegend, San Diego, CA, USA) stainings according to the manufacturer’s recommendations. For Oil Red O staining a 0.5% (w/v) stock solution of Oil Red O (Sigma-Aldrich, St. Louis, MO, USA) in isporopanol (> 99.8%) was filtered through Whatman paper. A working solution was prepared by mixing 6 parts of stock solution with 4 parts of water. Lipids were stained in freshly filtered working solution of Oil red O, cell nuclei in Mayer's Haematoxylin (Carl Roth, Karlsruhe, Germany). Atherosclerotic plaque formation was assessed investigating all cryosections including the cusps of the aortic valve (≥ 5 sections per animal) by an investigator blinded for the genotype. The mean plaque burden of every mouse was calculated and used for further analyses. CD11b and MOMA-2 stainings were analyzed by quantifying the positive area per total plaque area.

### Flow cytometry

The aorta of each mouse was used to obtain single-cell suspensions. Aortae were extensively flushed with phosphate buffered saline to remove blood leukocytes and then excised from aortic root to iliac bifurcation. However, a marginal contamination with blood cells cannot be ruled out. Perivascular tissue was carefully removed using a microscope (microdissection). Subsequently, aortae were minced with scissors and digested in collagenase I (450 U/ml), collagenase XI (125 U/ml), DNaseI (60 U/ml), and hyaluronidase (60 U/ml) (Sigma-Aldrich, St. Louis, MO, USA) at 37 °C and 750 rpm for 1 h for staining of myeloid cells as it has been described previously [31]. Unlike histology, flow cytometry does not allow to distinguish between intimal and adventitial leukocytes. Consequently, changes in plaque leukocyte numbers—assessed by flow cytometry—may not solely be driven by intimal leukocytes, but also by adventitial leukocytes.

Myeloid cell staining was carried out as described previously [31]. In brief, cells were first stained with mouse hematopoietic lineage markers covering phycoerythrin (PE) anti-mouse antibodies directed against B220 (clone RA3-6B2, BD Biosciences, Franklin Lakes, NJ, USA), CD90 (clone 53-2.1, BioLegend, San Diego, CA, USA), CD49b (clone DX5, BD Biosciences, Franklin Lakes, NJ, USA), Ly-6G (clone 1A8, BD Biosciences, Franklin Lakes, NJ, USA), NK1.1 (clone PK136, BioLegend, San Diego, CA, USA), and Ter-119 (clone TER-119, BD Biosciences, Franklin Lakes, NJ, USA). Subsequently, a second staining was carried out including CD45.2 (clone 104, BD Biosciences, Franklin Lakes, NJ, USA), CD11b (clone M1/70, BD Biosciences, Franklin Lakes, NJ, USA), CD115 (cloneAFS98, BioLegend, San Diego, CA, USA), CD11c (clone HL3 BD Biosciences, Franklin Lakes, NJ, USA), F4/80 (clone BM8, BioLegend, San Diego, CA, USA), MHCII (clone M5/114.15.2, BioLegend, San Diego, CA, USA), and Ly6C (clone AL-21, BD Biosciences, Franklin Lakes, NJ, USA).

Neutrophils were identified as (CD90/B220/CD49b/NK1.1/Ter119)^low^ (CD45.2/CD11b)^high^ CD115^low^ Ly6G^high^ [31]. Monocytes were identified as (CD90/B220/CD49b/NK1.1/Ter119)^low^ CD11b^high^ (F4/80/CD11c)^low^ Ly-6C^high/low^ or (CD45.2/CD11b)^high^ Ly6G^low^ CD115^high^ Ly-6C^high/low^ [31]. Macrophages were identified as (CD90/B220/CD49b/NK1.1/Ter119)^low^ CD11b^high^ Ly6C^low/int^ Ly6G^low^ F4/80^high^ [31]. A viability dye was not routinely used in our stainings, because respective channels were already occupied by other dyes necessary to identify leukocyte subsets of interest. Hence, viable leukocytes were identified based on size/volume (forward scatter, FSC) and complexity/granularity (side scatter, SSC) in the very first gate. However, a marginal contamination with dead cells cannot be excluded.

To obtain purified neutrophils and monocytes for adoptive transfer experiments, cells were isolated from the bone marrow of Ubc-GFP donor animals using Ly6G-PE (127608, clone 1A8) and CD115-biotin (135508, clone AFS98, both BioLegend, San Diego, CA, USA) that allowed coupling to magnetic beads (anti-PE and streptavidin microbeads, 130-048-801 and 130–048-101, Miltenyi Biotec, Bergisch Gladbach, Germany) and separation of cells via magnetic-activated cell separation columns (130-042-401, Miltenyi Biotec, Bergisch Gladbach, Germany). Equal amounts of purified neutrophils and monocytes were injected i.v. into *ApoE*^−/−^*Svep1*^+/−^ or *ApoE*^−/−^ mice which were fed a high-cholesterol diet for 6 weeks. The aortae were harvested and analyzed as described above. The number of CD11b^high^GFP^high^ cells within the aortae was quantified using flow cytometry.

### Cloning of SVEP1 constructs

A vector containing the *SVEP1* open reading frame followed by a Myc-DDK-tag (NM_153366, RC214271; pCMV_SVEP1) was purchased from OriGene Technologies (Rockville, MD, USA). The p.D2702G amino acid change (pCMV_SVEP1_p.D2702G) was introduced using in vitro site-directed mutagenesis (QuikChange II XL; Agilent Technologies, Santa Clara, CA, USA) according to the supplier’s protocol and using the following mutagenesis primers: *SVEP1var_for*: 5′-GCC ATT CCA AGT TCC ACC TTC CTG GCA GAT CAG-3′, *SVEP1var_rev*: 5′- CTG ATC TGC CAG GAA GGT GGA ACT TGG AAT GGC-3′. Coding sequence of human SVEP1 was then cloned into a pcDNA-pDEST40 expression vector C-terminally tagged with a HA-tag (Life Technologies, Carlsbad, CA, USA). The expression plasmids were sequenced on both strands prior to transfection of eukaryotic cells.

### Overexpression of SVEP1 constructs and generating concentrated conditioned media

Human embryonic kidney (HEK) 293 E cells were seeded at a density of 6 million cells per 100 mm cell culture dish 24 h prior to transfection. FuGENE HD Transfection Reagent (Promega, Madison, WI, USA) was used for transfection according to the manufacturer’s recommendation. Medium was exchanged for 10 ml serum-free DMEM just before transfection. Per dish, 5 µg of plasmid (pcDNA-DEST40_SVEP1-HA, pcDNA-DEST40_SVEP1_p.D2702G-HA) were incubated with 20 µl FuGENE HD Transfection Reagent and 800 µl Opti-MEM I Reduced Serum Medium (Gibco by Life Technologies, Carlsbad, CA, USA) for 15 min at room temperature. Afterwards, DNA-FuGENE complexes were added to the cells. 48 h after transfection, conditioned medium was collected and centrifuged at 4800*g* for 20 min to eliminate suspended cells. After filtering through 22 µm PES filter (Carl Roth, Karlsruhe, Germany), the filtrate was concentrated in Amicon Ultra® 100 k MWCO filter units (Merck Millipore, Billerica, MA, USA) according to the manufacturer’s protocol to a final volume of 200 µl. To generate cell lysates, cells were washed with PBS and resuspended in RIPA buffer (Cell Signaling Technology, Danvers, MA, USA). After incubating for 30 min at − 80 °C and thawing on ice, cell suspensions were transferred to centrifugal tubes and sonicated for 3·30 s with intermittent 30 s incubations on ice. Lysates were subsequently centrifuged for 30 min at 20,000*g*.

### CXCL1 enzyme-linked immunosorbent assays

CXCL1 enzyme-linked immunosorbent assays (ELISA) were used to determine human (ab100530) and murine (ab216951; both Abcam, Cambridge, UK) CXCL1 protein levels in cell culture supernatant and plasma samples, respectively, according to the suppliers’ recommendations.

### Stimulating HUVEC with SVEP1 or SVEP1 and interleukin 1β

HUVEC (PromoCell, Heidelberg, Germany) were seeded at a density of 200,000 cells in 12-well cell culture plates. 24 h after seeding the cells were stimulated with concentrated conditioned media containing either SVEP1-HA, SVEP1-D2702G-HA or mock control. After 24 h, cells were harvested for RNA isolation. To investigate interleukin 1β-induced *CXCL1* expression, *HUVEC* were seeded at a density of 160,000 cells in 24-well cell culture plates. Two h after seeding the cells were stimulated with a final concentration of 12.5 ng/µl recombinant human interleukin 1β (R&D systems, Minneapolis, MN, USA) and concentrated conditioned media either containing SVEP1 or mock control. After 24 h, supernatants were collected for subsequent analyses. Samples were frozen in liquid nitrogen and stored at − 80 °C.

### Phenome-wide association study of SVEP1 variants in UK Biobank

Phenome-wide association analysis for rs111245230 was conducted using the UK Biobank dataset. We integrated the International Classification of Disease ICD9, 10, OPCS-4 (Office of Population, Censuses and Surveys: Classification of interventions and Procedures, version 4) and self-reported information to define individual condition with different phenotypes. Disease classification was used to better navigate through the data, and we grouped phenotypes into cardiovascular, endocrine, neurological, digestive, genito-urinary, musculoskeletal, respiratory, eye, cancer and others [11, 26]. Finally, we identified 64 phenotypes and grouped them into nine classes. We extracted the genotype for rs111245230 from the full UK Biobank imputed dataset on 487,406 participants and did a quality control to filter samples with high kinship coefficient (> 0.088) and more than ten putative third-degree relatives in the kinship table. Samples without clear disease definition were also removed during analysis. We used PLINK [7] to test the association between rs111245230 and each phenotype independently based on logistic regression with the additive genetic model. The adjustment of population stratification includes age, gender and the top two principal components. A Bonferroni corrected threshold (*p* < 0.0008) was used to determine significantly related phenotypes.

### Statistical analysis

Data distribution was assessed using the Kolmogorov–Smirnov test. Data were analyzed using Student’s/Welch’s unpaired/paired/one-column t test (in case of normally distributed data) or Mann–Whitney/Wilcoxon test (in case of not normally distributed data), as appropriate and indicated in the figure legends. *p* values < 0.05 were regarded as significant. GraphPad Prism version 8 for Mac OS X (GraphPad Software, La Jolla, CA, USA) was used.

## Results

### SVEP1 is expressed in vascular and metabolic tissues

**Fig. 1:**
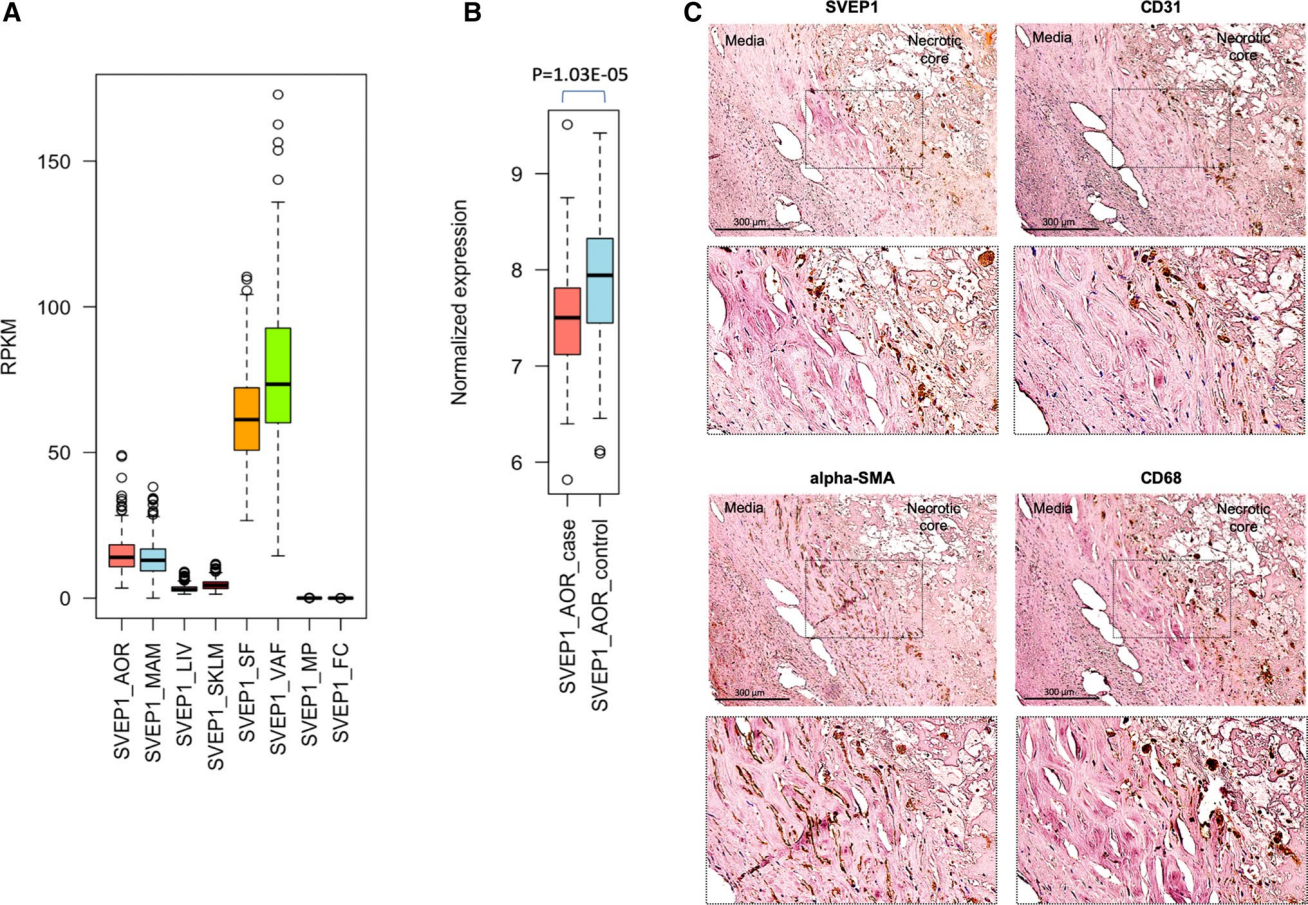
*SVEP1* expression in vascular and metabolic tissues **a** *SVEP1* is expressed in subcutaneous fat, visceral fat, aorta, and mammary artery. *SVEP1* expression is low in liver and skeletal muscle and its expression is extremely low in macrophage and foam cells. **b** *SVEP1* is differentially expressed in the aortae of CAD cases versus controls (*n* = 102 cases/79 controls). **c** Immunohistochemistry staining of atherosclerotic plaques from human carotid arteries using antibodies against SVEP1, CD31, alpha-SMA, and CD68. An overview image of the atherosclerotic plaque is shown in Suppl. Fig. S3. Sections of further atherosclerotic plaques staining positive for SVEP1 are depicted in Suppl. Fig. S4. *alpha-SMA*, alpha-smooth muscle actin; *AOR*, aorta; *FC*, foam cells; *LIV*, liver; *MAM*, mammary artery; *MP*, macrophages; *RPKM*, reads per kilobase million; *SF*, subcutaneous fat; *SKLM,* skeletal muscle; *VAF*, visceral fat

Currently, SVEP1’s role in CAD has not yet been demonstrated. To test whether SVEP1 is localized in vascular tissue, we explored relevant *SVEP1* expression in human tissues. Here, we evaluated *SVEP1* mRNA levels in RNA sequencing (RNAseq) data from the STARNET study which examined gene expression in seven metabolic and vascular tissues [14]. We found the highest *SVEP1* expression levels in adipose tissue (visceral adipose tissue and subcutaneous fat) and vessels (aorta and mammary artery) (Fig. 1a). Of note, these tissues also show highest SVEP1 expression levels in the GTEx database [16] (Suppl. Fig. S2). The strongest difference in *SVEP1* expression was detected in aortae of patients with CAD as compared to those from controls with lower levels in cases (Fig. 1b). In addition to mRNA, we next examined whether also SVEP1 protein is detectable in vascular tissues. Using immunohistochemistry in carotid artery plaques from patients undergoing carotid artery endarterectomy, we detected SVEP1 in proximity to smooth muscle cells (SMC), endothelial cells (EC), and CD68-positive cells as monocytes/macrophages/secretory VSMC (using alpha-smooth muscle actin, CD31, and CD68 as markers respectively) (Fig. 1c; Suppl. Figs. S3, 4). These data indicate that SVEP1 is present in the vasculature with reduced levels in patients with atherosclerosis, i.e. CAD cases.

### Endothelial cells and smooth muscle cells represent a major cellular source of SVEP1 in the vasculature

**Fig. 2:**
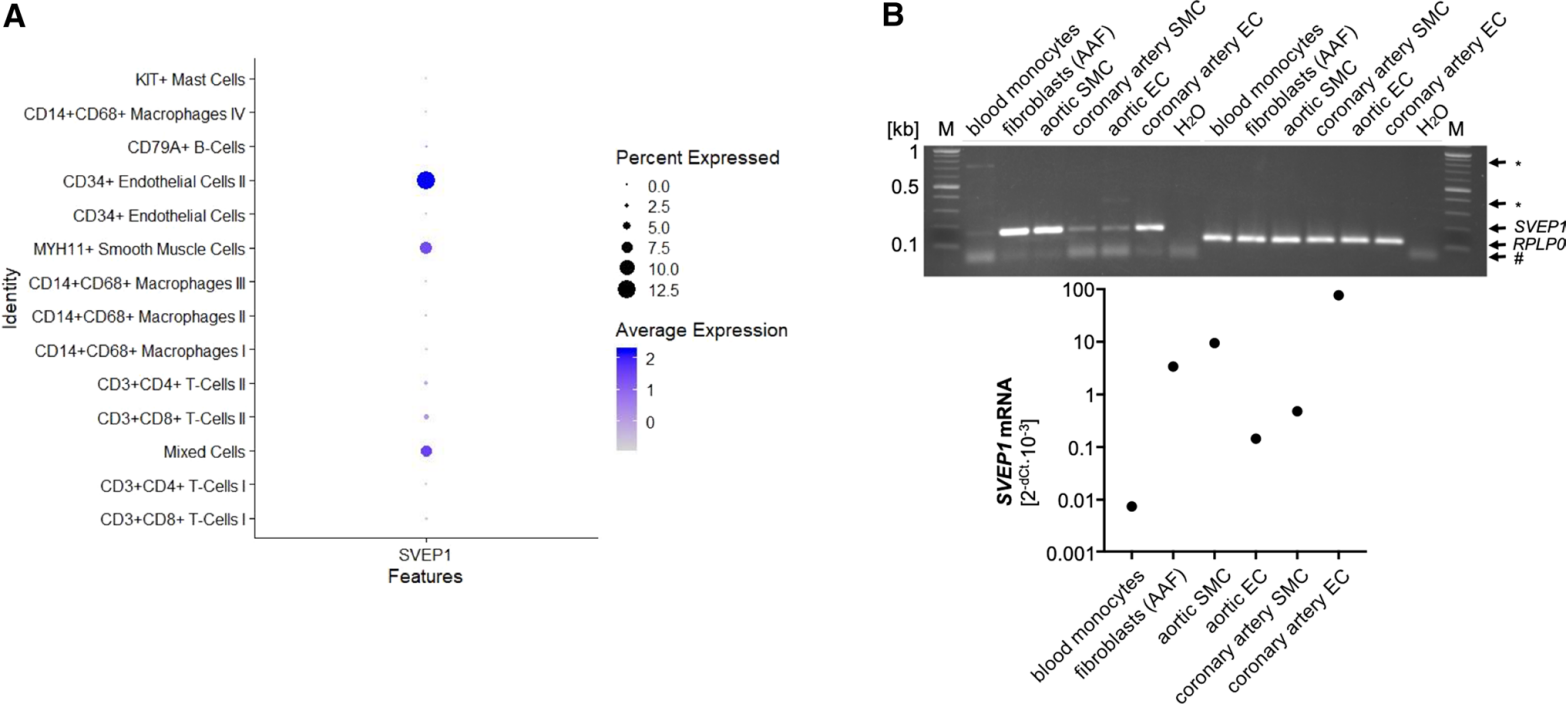
*SVEP1* is expressed in endothelial cells and smooth muscle cells in atherosclerotic plaques. **a** Single-cell RNA sequencing of atherosclerotic plaques from human carotid arteries reveals the highest *SVEP1* expression in endothelial cells (cluster II, e.g. expressing *VWF* and *EDN1*). **b** *SVEP1* expression in vascular cell types. *Unspecific band(s); ^#^primer dimer(s). *RPLP0* and *GAPDH* served as housekeeping genes in gel and qPCR analyses, respectively. Abbreviations: *AAF*, aortic adventitial fibroblasts; *EC*, endothelial cell(s); *M*, Marker; *SMC*, smooth muscle cell(s). Monocytes are isolated classical monocytes from peripheral blood

As SVEP1 is an ECM protein and hence released from cells, immunohistochemistry is less suitable to precisely determine the cellular sources of SVEP1. We thus examined *SVEP1* expression in single cell RNAseq (scRNAseq) data from human carotid artery plaques to determine the cellular source of SVEP1 in the vessel wall. We found EC and vascular SMC had the highest expression levels (Fig. 2a; Suppl. Fig. S5). In addition, we analyzed *SVEP1* mRNA in primary fibroblasts, SMC, and EC in cell culture, as well as in classical monocytes isolated from human whole blood. Strong expression of *SVEP1* was detected in primary fibroblasts, SMC, and EC, whereas blood monocytes showed only weak expression (Fig. 2b). Taken together, these data indicate that SVEP1 is mainly produced by EC and vascular SMC within vascular and atherosclerotic tissue.

### Svep1 knockdown promotes atherosclerotic plaque formation

**Fig. 3:**
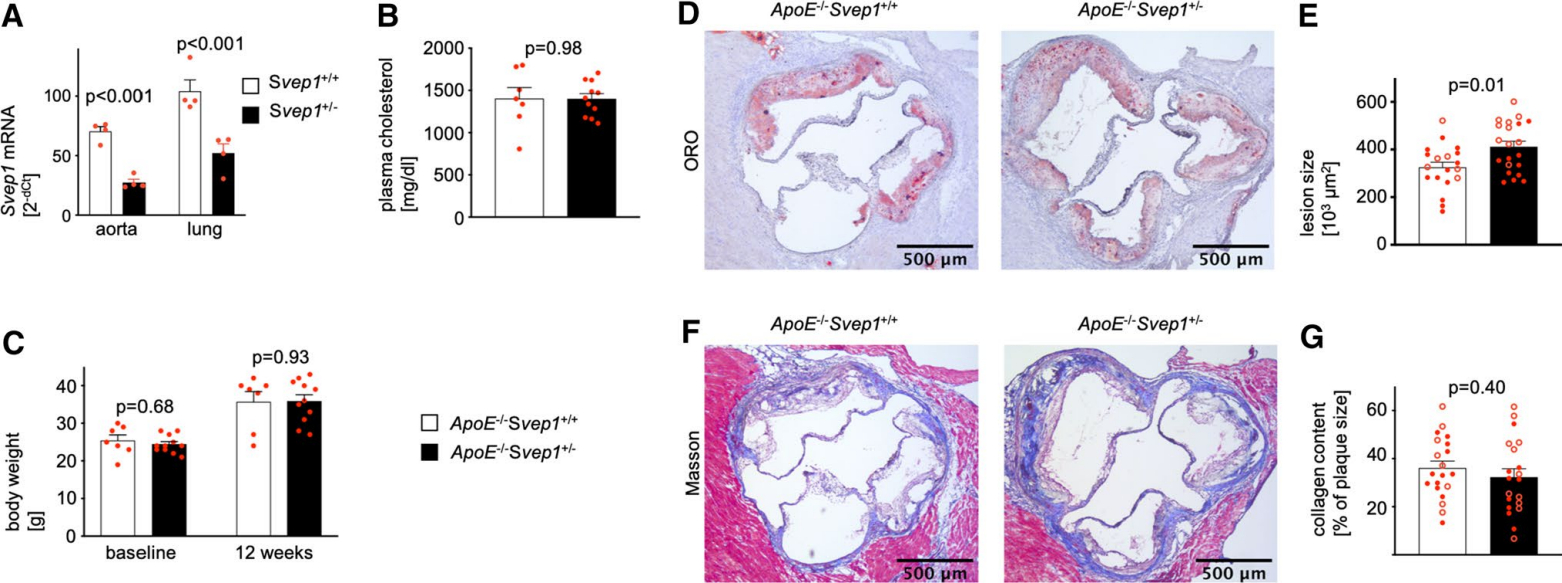
Svep1 knockdown increases atherosclerotic plaque burden. **a** Quantification of *Svep1* mRNA levels in lung and aorta of *Svep1*^+/+^ and *Svep1*^+/–^ mice. *Gapdh* served as housekeeping gene. **b** Plasma cholesterol levels after western diet (WD). **c** Bodyweight of *ApoE*^−/−^*Svep1*^+/+^ and *ApoE*^−/−^*Svep1*^+/–^ mice at baseline and after WD. **d**, **e** Atherosclerotic plaques in aortic roots after WD in *ApoE*^−/−^*Svep1*^+/+^ and *ApoE*^−/−^*Svep1*^+/–^ mice. **f**, **g** Collagen content of atherosclerotic plaques in the aortic root after WD in *ApoE*^−/−^*Svep1*^+/+^ and *ApoE*^−/−^*Svep1*^+/–^ mice (Masson's trichrome). Data are mean and s.e.m. Unpaired *t* test (**a**–**c**, **e**, **g**). **e**, **g** Filled circles, male mice; unfilled circles, female mice. *ORO*, Oil Red O

To better explore the impact of SVEP1 on atherosclerosis, we crossbred *ApoE*^−/−^ mice with *Svep1*^+/+^ or *Svep1*^+/−^ mice and hence generated atherosclerosis-prone mice with either normal (*ApoE*^−/−^*Svep1*^+/+^) or reduced (*ApoE*^−/−^*Svep1*^+/−^) wildtype Svep1 levels. Complete Svep1 deficiency (*Svep1*^−/−^) is known to be lethal [23]. *Svep1*^+/−^ mice did not show an obvious phenotype and behaved normally as described previously [23]. Furthermore, *Svep1*^+/−^ mice have not been reported to show cardiovascular phenotypes under baseline conditions (Suppl. Fig. S6). Compared to *Svep1*^+/+^ mice, *Svep1*^+/−^ mice displayed reduced *Svep1* mRNA levels in lungs [52.2 ± 7.7 vs. 104.2 ± 9.6 (2^−dCt^), *p* < 0.001] and aortae [27.5 ± 2.7 vs. 70.4 ± 4.0 (2^−dCt^), *p* < 0.001] (Fig. 3a). To investigate the impact of Svep1 on atherosclerotic plaque formation, 8 weeks old *ApoE*^−/−^*Svep1*^+/+^ and *ApoE*^−/−^*Svep1*^+/−^ mice were fed a western diet (WD) for 12 weeks followed by histological analysis of the aortic root. Total plasma cholesterol levels rose similarly in both genotypes [*ApoE*^−/−^*Svep1*^+/−^ 1,401 ± 61.5 vs. *ApoE*^−/−^*Svep1*^+/+^ 1404 ± 130.2 (mg/dl), *p* = 0.98; Fig. 3b]. Further, both genotypes displayed a consistent increase in body weight during WD feeding [*ApoE*^−/−^*Svep1*^+/−^ vs. *ApoE*^−/−^*Svep1*^+/+^; baseline: 24.5 ± 0.7 vs. 25.4 ± 1.5 (g), *p* = 0.68; after diet: 35.9 ± 1.7 vs. 35.7 ± 2.7 (g), *p* = 0.93; Fig. 3c]. Atherosclerotic plaques were larger in *ApoE*^−/−^*Svep1*^+/−^ mice compared to *ApoE*^−/−^*Svep1*^+/+^ mice as determined by Oil Red O staining [411.8 ± 22.9 vs. 325.1 ± 22.0 (10^3^ µm^2^), *p* = 0.01; Fig. 3d, e). Collagen content was similar in *ApoE*^−/−^*Svep1*^+/−^ compared to *ApoE*^−/−^*Svep1*^+/+^ mice [32.3 ± 3.5 vs. 36.1 ± 2.9 (% of plaque), *p* = 0.40; Fig. 3f, g].

### Svep1 knockdown increased leukocyte recruitment to the vascular wall in vivo

**Fig. 4:**
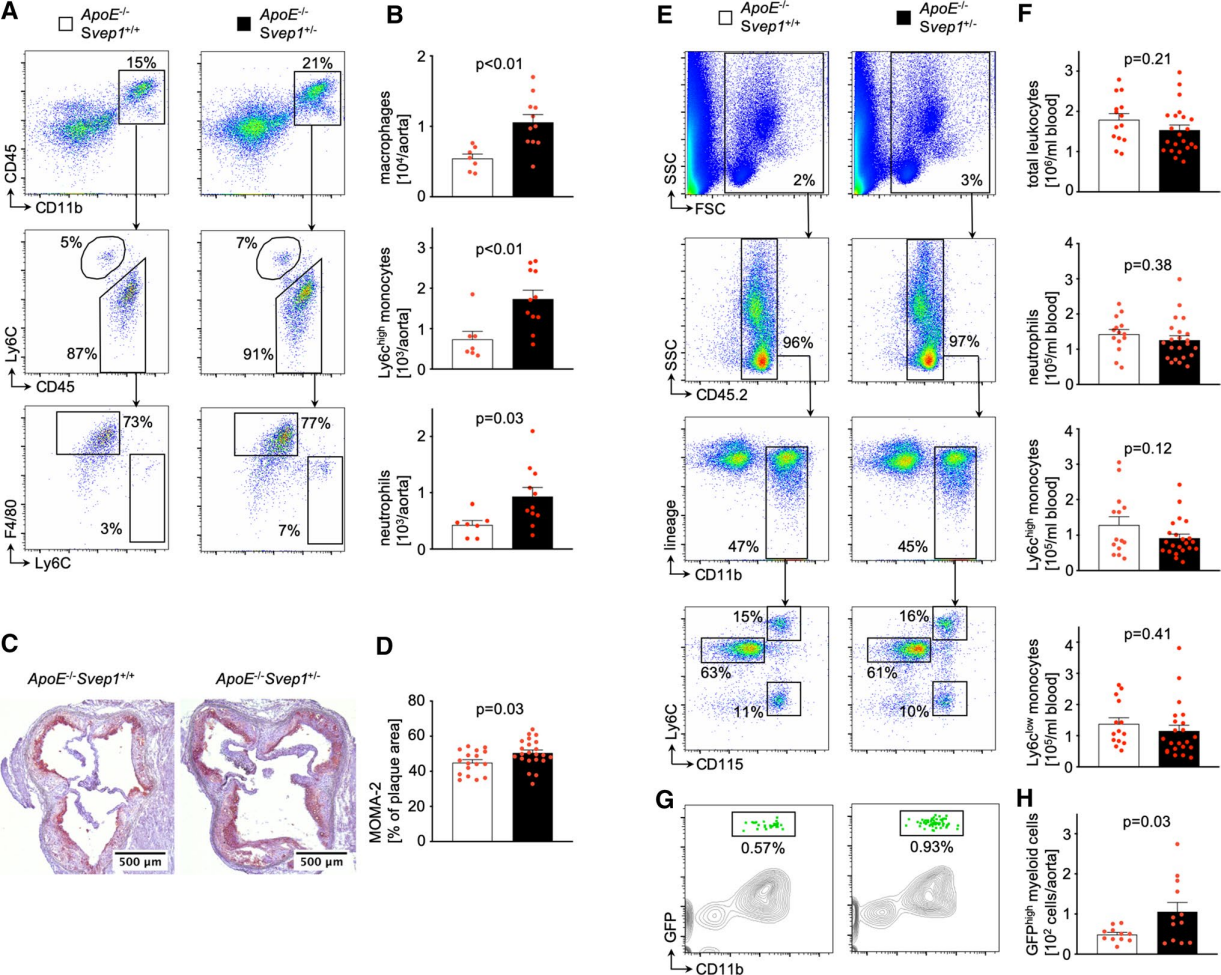
Svep1 knockdown increases vascular inflammation. **a**, **b** Flow cytometric gating (**a**) and quantification (**b**) of plaque leukocyte subsets in *ApoE*^−/−^*Svep1*^+/+^ and *ApoE*^−/−^*Svep1*^+/–^ mice on WD for 12 weeks. **c**, **d** Monocyte and macrophage staining (Monocyte + Macrophage antibody (MOMA-2)) of aortic roots from *ApoE*^−/−^*Svep1*^+/+^ and *ApoE*^−/−^*Svep1*^+/–^ mice. **e**, **f** Flow cytometric gating (**e**) and quantification (**f**) of blood leukocyte subsets in *ApoE*^−/−^*Svep1*^+/+^ and *ApoE*^−/−^*Svep1*^+/–^ mice on WD for 12 weeks. Numbers next to gates indicate population frequencies [%]. Unpaired Student’s *t* test. **g**, **h** Detection (**g**) and quantification (**h**) of adoptively transferred GFP^high^ cells in aortae of *ApoE*^−/−^*Svep1*^+/+^ and *ApoE*^−/−^*Svep1*^+/–^ mice on WD for 6 weeks. Welch’s *t* test. Data are mean and s.e.m. Each symbol represents one mouse

Since vascular inflammation is a major contributor to plaque initiation and progression, we assessed plaque leukocyte accumulation in aortae of *ApoE*^−/−^*Svep1*^+/+^ and *ApoE*^−/−^*Svep1*^+/–^ mice using flow cytometry. Plaque macrophage [10,573 ± 1,110 vs. 5439 ± 635 (cells), *p* < 0.01], Ly6C^high^ monocyte [1735 ± 219 vs. 736 ± 199 (cells), *p* < 0.01], and neutrophil [933 ± 161 vs. 429 ± 79 (cells), *p* = 0.03] numbers increased in *ApoE*^−/−^*Svep1*^+/–^ over *ApoE*^−/−^*Svep1*^+/+^ mice (Fig. 4a, b). In accordance, immunohistochemical stainings for leukocytes (Suppl. Fig. S7) as well as monocytes and macrophages confirmed higher numbers of inflammatory immune cells inside atherosclerotic plaques (Fig. 4c, d). Of note, we also observed higher numbers of non-classical (Ly6C^low^) monocytes/macrophages in plaques of *ApoE*^−/−^*Svep1*^+/–^ mice (Suppl. Fig. S8). Whether this population derives from recruited Ly6C^low/high^ blood monocytes or from Ly6C^low^ lesional macrophages is currently poorly understood [5, 6]. We next explored how plaque leukocytes increased in number in *Svep1* knockdown mice. Numbers of total blood leukocytes [1,534,545 ± 127,069 vs. 1,791,429 ± 153,879 (cells/ml blood), *p* = 0.21], blood neutrophils [251,544 ± 25,520 vs. 285,573 ± 27,073 (cells/ml blood), *p* = 0.38], blood Ly6C^high^ monocytes [91,985 ± 11,156 vs. 128,917 ± 23,614 (cells/ml blood), *p* = 0.12], and blood Ly6C^low^ monocytes [115,241 ± 18,417 vs. 138,333 ± 19,283 (cells/ml blood), *p* = 0.41] were comparable between *ApoE*^−/−^*Svep1*^+/–^ and *ApoE*^−/−^*Svep1*^+/+^ mice (Fig. 4e, f), thereby indicating that Svep1 deficiency did not impact inflammatory leukocyte supply. Thus, we tested whether Svep1 might impact inflammatory blood leukocyte recruitment to atherosclerotic plaques.

To address this, we retrieved green fluorescence protein positive (GFP^high^) monocytes and neutrophils from naïve transgenic mice (*Ubc-GFP)* and adoptively transferred these cells into either *ApoE*^−/−^*Svep1*^+/–^ or *ApoE*^−/−^*Svep1*^+/+^ mice which were fed a WD for 6 weeks (Suppl. Fig. S9). Using flow cytometry 24 h after the transfer, we detected more GFP^high^ myeloid cells inside plaques in *ApoE*^−/−^*Svep1*^+/–^ in comparison to *ApoE*^−/−^*Svep1*^+/+^ mice [105.8 ± 80 vs. 48.9 ± 18 (cells), *p* = 0.03] indicating that SVEP1 affects leukocyte recruitment (Fig. 4g, h).

### SVEP1 silences CXCL1 expression in endothelial cells

**Fig. 5:**
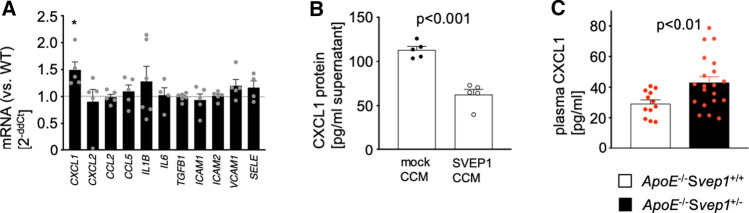
SVEP1 alters endothelial cell *CXCL1* expression. **a** Expression of inflammatory cytokines, chemokines, and cell adhesion molecules, as shown by fold changes after incubation of HUVEC with SVEP1_p.D2702G compared to SVEP1 are displayed. Data are mean and s.e.m. One-sample *t* test. *Denotes *p* < 0.05. **b** Secreted CXCL1 decreases after incubation of interleukin 1β-primed HUVEC with concentrated media containing SVEP1 in five experiments. Data are mean and s.e.m. Unpaired *t* test. **c** Plasma Cxcl1 levels in *ApoE*^−/−^*Svep1*^+/+^ and *ApoE*^−/−^*Svep1*^+/–^ mice. Welch’s *t* test. Data are mean and s.e.m. *WT*, wild type

Leukocyte recruitment is a process in which EC and circulating leukocytes act in concert to mediate rolling, adhesion, and transmigration [15]. Hence, we tested whether SVEP1 impacts endothelial cell phenotypes. HUVEC, which exhibit weak endogenous *SVEP1* expression (Suppl. Fig. S10), were incubated with either wild-type SVEP1 or missense SVEP1 (SVEP1_p.D2702G). While investigating a panel of inflammatory transcripts, we found that incubation with SVEP1_p.D2702G, i.e. mutant SVEP1 without wild-type SVEP1, led to an increase in chemokine (C-X-C motif) ligand 1 (*CXCL1*) mRNA levels [+ 49.2 ± 0.15 (%), *p* = 0.03; Fig. 5a] compared to wild-type SVEP1. CXCL1 is a strong chemoattractant for both neutrophils and monocytes [15]. As the CAD-associated risk variant—which leads to a lack of wild-type SVEP1 in patients—elevated *CXCL1* expression and Svep1-deficient mice showed enlarged plaque sizes, we wondered whether SVEP1 directly impacts endothelial *CXCL1* expression. After incubation with concentrated conditioned media containing wild-type SVEP1, CXCL1 protein levels in EC supernatant decreased when exposed to SVEP1 compared to control (Fig. 5b). In line, we found elevated plasma CXCL1 levels in *ApoE*^−/−^*Svep1*^+/−^ compared to *ApoE*^−/−^*Svep1*^+/+^ mice [43.0 ± 3.8 vs. 29.2 ± 2.4 (pg/ml), *p* < 0.01; Fig. 5c].

In addition to the chemokine CXCL1, we also detected lower *SELE* expression in EC when exposed to wild-type SVEP1 compared to control (Suppl. Fig. S11). *SELE* encodes E-selectin, a cell adhesion molecule that together with chemokines is pivotal in mediating leukocyte recruitment [15]. Our data indicate that wild-type SVEP1 controls EC leukocyte recruitment capacities and that the CAD risk variant SVEP1_p.D2702G mimics reduced intact wild-type SVEP1 levels.

### Phenome-wide association study of SVEP1

**Fig. 6:**
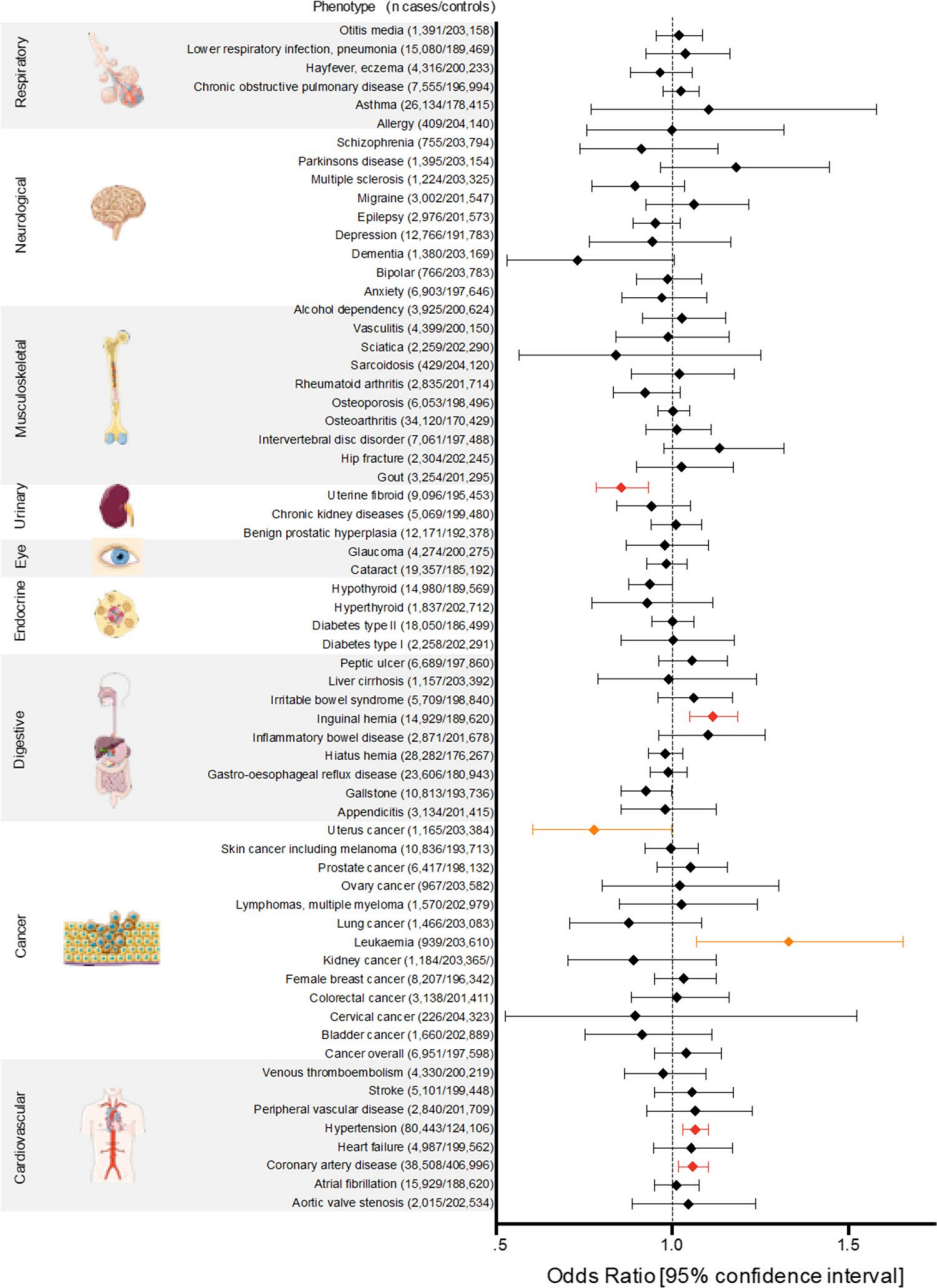
Phenotype-wide association study of SVEP1_p.D2702G. The CAD risk variant was associated with an increased risk of hypertension and inguinal hernia but a reduced risk of uterine fibroid after correction for multiple testing (red). Furthermore, there was a nominally significant trend toward higher risk of leukemia, but reduced risk for uterine cancer (orange). Odds ratios and unadjusted p values are depicted in Suppl. Table S2

Phenome-wide association studies are powerful tools to evaluate whether either inhibition or activation of genes might beneficially or deleteriously affect other phenotypes. Therefore, we performed a *SVEP1* phenome-wide association study (PheWAS) in 445,504 individuals included in UK Biobank [21]. After Bonferroni correction, rs111245230 genotype was, in addition to CAD, associated with an increased risk of hypertension (OR 1.07, 95% CI 1.03–1.10, *p*_adj_ = 0.02) and inguinal hernia (OR 1.12, 95% CI 1.05–1.19, *p*_adj_ = 0.03) but a reduced risk of uterine fibroids (OR 0.86, 95% CI 0.78–0.93, *p*_adj_ = 0.03) (Fig. 6, Suppl. Table S1).

## Discussion

Knowledge of the molecular and cellular functions of SVEP1 is limited. Besides its role in lymphatic vessel formation [17, 23], SVEP1 has been investigated in the context of septic shock and endotoxinemia [25, 34]. The fact that homozygous loss of *Svep1* is lethal [17, 23] suggests an important role in developmental processes. Our data provide a first insight into the role of SVEP1 in atherosclerosis. In a series of in vitro and in vivo experiments, we explored the role of the recently identified CAD gene. We found that SVEP1 was detectable in human atherosclerotic plaques and produced by various vascular wall cell types, including vascular SMC but particularly EC. Under proatherogenic conditions, *ApoE*^−/−^ mice with Svep1 haploinsufficiency developed larger atherosclerotic plaques compared to *ApoE*^−/−^ mice with normal Svep1 levels. In addition, plaque leukocytes were more numerous in *ApoE*^−/−^*Svep1*^+/−^ compared to *ApoE*^−/−^*Svep1*^+/+^ mice. In adoptive transfer experiments, we demonstrated that plaque leukocyte expansion is a consequence of enhanced leukocyte recruitment in the presence of reduced Svep1 levels. EC are critically involved in leukocyte recruitment under proatherogenic conditions [15, 35]. This and the fact that SVEP1 is located extracellularly led to the hypothesis that it might influence EC phenotypes. To get a first insight into whether the CAD risk variant SVEP1_p.D2702G (like *Svep1*^+/−^ mice, carriers of this missense variant also lack wild-type SVEP1) leads to decreased SVEP1 function, we analyzed EC phenotypes after incubation with either SVEP1 or SVEP1_p.D2702G. Adding SVEP1_p.D2702G was associated with increased expression of the chemokine *CXCL1* and the adhesion molecule *SELE* suggesting wild-type SVEP1 plays a rather atheroprotective role that was altered in the presence of the SVEP1_p.D2702G variant. In CAD cases, we further found reduced *SVEP1* mRNA levels compared to controls. In addition, our data show that SVEP1 exerts regulating effects on EC resulting in decreased *CXCL1* and *SELE* expression in the presence of higher SVEP1 levels in vitro. Both CXCL1 and E-selectin mediate leukocyte recruitment from blood to plaques and hence play a crucial role in the early stages of atherosclerosis when dysfunctional EC secrete chemokines/upregulate adhesion molecules and thereby induce monocyte and neutrophil adhesion [15, 37]. In particular, EC-derived CXCL1 has been shown to promote leukocyte recruitment under proatherogenic conditions [37]. SVEP1 led to a dose-dependent decrease in CXCL1 protein levels, demonstrating that SVEP1 likely helps regulate EC phenotypes. This hypothesis is further supported by the finding that mice with a heterozygous loss of *Svep1* also displayed elevated Cxcl1 plasma levels under proatherogenic conditions. Also, the observed increased atherosclerotic plaque formation and more numerous neutrophils, Ly6C^high^ monocytes, and macrophages inside plaques of *ApoE*^−/−^*Svep1*^+/−^ compared to *ApoE*^−/−^*Svep1*^+/+^ mice accord with the downstream effects of CXCL1 and E-selectin in atherosclerosis [15, 37]. Of note, we previously identified SVEP1 as a target of the protease ADAMTS-7 [18]. The *ADAMTS7* gene has been linked to atherosclerotic plaque formation in humans [8, 30, 33] and mice [2]. While the molecular and cellular mechanisms involving ADAMTS-7 in atherosclerosis have not yet been elucidated, SVEP1 degradation may be involved.

Drug targets identified by genetic analyses reportedly have higher success rates in the drug development pipeline [27]. Our findings raise the question of whether increasing wild-type SVEP1 protein levels, e.g. via inhibiting its degradation, might also have beneficial effects regarding other disorders and phenotypes. To explore this, we conducted a PheWAS in UK Biobank. We replicated the association between the CAD risk variant and hypertension; this risk variant was also associated with a higher risk of inguinal hernia and trend for leukemia. When evaluating this concept’s therapeutic potential, it is important to also consider possible detrimental effects. The CAD risk allele was associated with a reduced risk of uterine fibroids and trend for uterine cancer. While uterine fibroids are considered benign, familial prevalence of fibroids has been linked with a higher risk of uterine cancer [28]. However, reliable data are scarce. While modulating SVEP1 may be considered as a novel treatment strategy its consequences on other phenotypes, especially malignancies, nevertheless warrant further investigation.

**Fig. 7:**
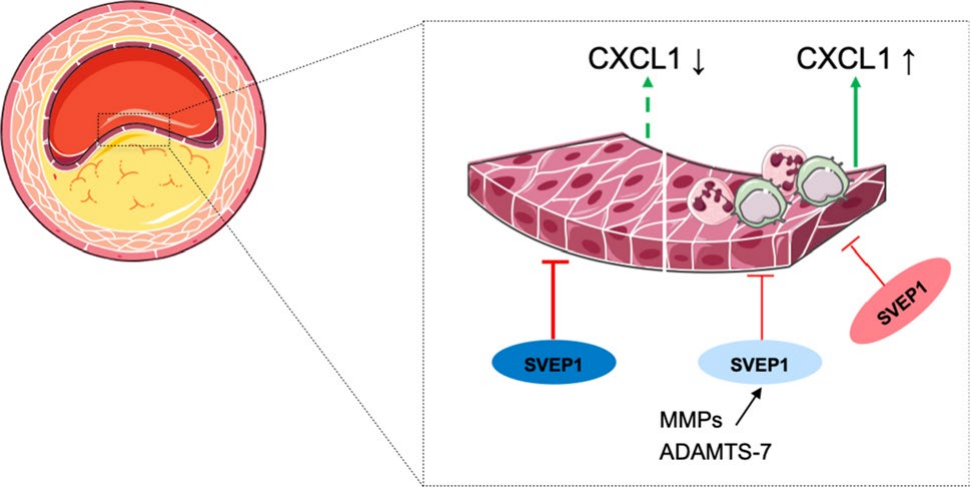
Schematic illustration of the role of SVEP1 and EC in atherosclerosis. In the presence of SVEP1 (blue), *CXCL1* expression is silenced. After SVEP1 degradation (light blue) or in the presence of SVEP1_p.D2702G (red), CXCL1 expression and secretion are increased, resulting in higher recruitment of inflammatory cells

In summary, we hypothesize that SVEP1 which is produced by vascular cell types and secreted to the ECM of the vascular wall acts as a silencing factor on EC activity by reducing the expression of chemokines and cell adhesion molecules, e.g., CXCL1 and E-selectin. If wild-type SVEP1 protein levels are reduced, e.g. via degradation by MMPs, or in the presence of mutant SVEP1 (SVEP1_p.D2702G missense variant), CXCL1 and E-selectin expression rises leading to enhanced inflammatory cell influx. Figure 7 displays this hypothesized functional cascade.

Whereas in this study, we postulate an atheroprotective effect of SVEP1 on EC, we cannot exclude further functions of SVEP1, particularly in other cell types. Specifically, the CAD risk variant has been shown to be associated with slight effects on blood pressure [24]. As SVEP1 is expressed in SMC, it might also influence SMC phenotypes. However, the association with blood pressure could not explain the risk variant’s association with CAD [24]. Furthermore, the role of SVEP1 in silencing CXCL1 and E-selectin expression by EC is independent of blood pressure since experiments were performed under no-flow, static conditions. Nevertheless, further effects, e.g. lower blood pressure in the presence of wild-type SVEP1, might provide additional atheroprotection. We also observed SVEP1 expression in plaque areas that also stained positive for the macrophage marker CD68. Although isolated blood monocytes only weakly express *SVEP1*, other cell types might also produce SVEP1 and thus influence plaque monocyte/macrophage phenotypes.

## Study limitations

Our study has several limitations. First, we do not know how SVEP1 mediates its effects on EC on a molecular level. ECM proteins have diverse effects on adjacent cell types that may surpass the known interaction with integrin α9β1. Second, we investigated a heterozygous knockout of *Svep1* which might not fully replicate the effects of the human risk variant since loss of intact wild-type SVEP1 may vary. However, we detected reduced *SVEP1* mRNA levels in vascular tissues from patients with CAD, as compared to controls, an event reflected by the mouse model we used. Third, and most importantly, findings from in vitro experiments do not necessarily reflect in vivo situations. In most in vitro approaches, the environment that naturally surrounds cells—in our case the ECM—is missing. Consequently, in vitro assays are somewhat artificial. We found that loss of SVEP1 leads to increased CXCL1 levels, both in vitro and in vivo*.* This indicates that at least with respect CXCL1 release, our in vitro data resemble our in vivo findings. It is of course possible that soluble SVEP1 exerts further in vivo effects that are not addressed in this study.

## Electronic supplementary material

Below is the link to the electronic supplementary material.

Supplementary file1 (DOCX 18387 KB)

## Data Availability

All data are presented in the text and the Supplemental Material.
