# Supplementary material for: Functional investigation of the coronary artery disease gene SVEP1
Source: Basic Res Cardiol. 2020 Nov 13;115(6):67. doi: 10.1007/s00395-020-00828-6 (PMC7666586; doi:10.1007/s00395-020-00828-6)
Supplement: Supplementary file 1 — Supplementary file1 (DOCX 18387 KB) [file 395_2020_828_MOESM1_ESM.docx]

**Functional investigation of the coronary artery disease gene *SVEP1***

**Supplemental Material**

**Supplemental Methods**

*Human carotid artery plaque specimen and single cell RNA sequencing*

Atherosclerotic plaques were obtained from 14 male and 4 female patients undergoing a (primary) carotid endarterectomy (CEA) procedure. All plaque specimens were included in the Athero-Express Biobank Study (AE, www.atheroexpress.nl), an ongoing biobank study at the University Medical Centre Utrecht (UMCU) [9]. Single cell RNA sequencing in these samples and further information has been published by Depuydt *et al*. [3]. Time between surgical removal and plaque processing did not exceed 10 min. The remainder of the plaque washed in RPMI and minced into small pieces with a razor blade. The tissue was then digested in RPMI 1640 containing 2.5 mg/mL Collagenase IV (ThermoFisher Scientific, Waltham, MA, USA), 0.25 mg/mL DNAse I (Sigma-Aldrich, St. Louis, MO, USA), 2.5 mg/mL Human Albumin Fraction V (MP Biomedicals, Illkirch-Graffenstaden, France) and 1 mM Flavopiridol (Selleckchem, Houston, TX, USA) at 37°C for 30 min. Subsequently, the plaque cell suspension was filtered through a 70 µm cell strainer and washed with RPMI 1640. Cells were kept in RPMI 1640 with 1% Fetal Calf Serum until subsequent staining for fluorescence-activated cell sorting. Single cell suspensions were stained with Calcein AM and Hoechst (ThermoFisher Scientific, Waltham, MA, USA) in PBS supplemented with 5% Fetal Bovine Serum (FBS) and 0.2% ethylenediaminetetraacetic acid (EDTA) for 30 minutes at 37°C. After staining, cells were washed and filtered through a 70 µm FlowMi cell strainer (SP Scienceware, Warminster, PA, USA). Viable cells, positive for both Calcein AM and Hoechst, were sorted using the Beckman Coulter MoFlo Astrios EQ. Using a Mosquito® HTS (TTP Labtech) 384 wells plates were filled with 50nL mineral oil containing CELseq2-primers, spike-ins and dinucleotide triphosphates (dNTPs). Viable cells were sorted one cell per well into these 384 wells plates and immediately frozen at -80°C until further processing. First, cDNA was constructed using the SORT-seq protocol95. In short, cells were lysed for 5 min at 65oC and subsequently reverse transcription and second strand mixes were added using the Nanodrop II liquid handling platform (GC biotech, Waddinxveen, Netherlands). Next, cells were pooled in one library and the aqueous phase was separated from the oil phase, followed by in vitro transcription (IVT). A library was formed using the CEL-Seq2 protocol [6]. Primers consisted of a 24 bp polyT stretch, a 64bp random molecular barcode (UMI), a cell-specific 8bp barcode, the 5′ Illumina TruSeq small RNA kit adaptor and a T7 promoter. For sequencing, TruSeq small RNA primers (Illumina, San Diego, CA, USA) were added to the libraries and sequenced paired end at 75 bp read length using Illumina NextSeq 500. HTseq Single-cell sequencing data was processed as described previously [8]. Analyses were performed in a R 3.5 environment using Seurat (version 2.3.4 and 3.0) [2]. Prior to processing, reads were filtered for mitochondrial and ribosomal genes, MALAT1, KCNQ1OT1, UGDH-AS1, and EEF1A. In order to omit doublets and low quality cells, only cells expressing between 500 and 10.000 genes and genes expressed in at least 3 cells were used for further analysis. The data was log-normalized and scaled with the exclusion of unique molecular identifiers (UMIs). Top variable genes for all samples were used to combine samples into one object using seurat function RunMultiCCA(), after which samples were aligned using AlignSubspace() with reduction.type=CCA and grouping.var=”plate”. Subsequently, canonical correlation analysis (CCA) reduction was performed with a resolution of 1.2 for 15 dimensions to identify clusters and to perform t-distributed stochastic neighbor embedding (tSNE). Cell types were assigned to cell clusters by evaluating gene expression of individual cell clusters using differential gene expression (Wilcoxon rank sum test) and analysis with SingleR [1] against BLUEPRINT [7] reference data. Sub-clustering of identified cell clusters was performed using CCA with a resolution of 0.9 or 1.5 for 15 dimensions. Downstream analysis of initial and sub-clusters was performed in a similar manner.

*Primary cells*

Monocytes were isolated from whole blood from one healthy individual using a combined Ficoll-Paque density gradient and a CD14 magnetic bead separation approach. In brief, 24 ml of peripheral venous blood were collected into BD Vacutainer CPT tubes with sodium citrate (BD Biosciences, Franklin Lakes, NJ, USA). Tubes were centrifuged at 1,600 g for 30 min at room temperature. Peripheral blood mononuclear cells (PBMC) were resuspended in plasma and pooled from three tubes. Tubes were centrifuged at 300 g for 15 min at 4° C. Pellets were resuspended in 160 µl magnetic-activated cell sorting (MACS) buffer (500 ml phosphate buffered saline supplemented with 2.5 mg bovine serum albumin (BSA fraction V powder, fatty acid free, Thermo Fisher Scientific, USA) and 2 ml ethylenediamine tetraacetic acid (EDTA, 0.5 M, Sigma, USA) with 40 µl of MACS CD14 beads (130-050-201, Miltenyi Biotec, Bergisch Gladbach, Germany). Cells were incubated for 15 min and centrifuged at 300 g for 10 min at 4°C. Cell pellets were resuspended in 500 µl of MACS buffer and applied to LS Columns (Miltenyi Biotec, Bergisch Gladbach, Germany) using a QuadroMACS Seperation Unit (Miltenyi Biotec, Bergisch Gladbach, Germany) for positive selection. Cells were eluted, centrifuged at 300 g for 10 min at 4°C and resuspended in cells QIAzol (1,500,000 cells/300 µl; Qiagen, Hilden, Germany).

Human coronary artery smooth muscle cells (HCASMC), human aortic adventitial fibroblasts (HAAF), human aortic endothelial cells (HAEC), human aortic smooth muscle cells (HASMC), human coronary artery endothelial cells (HCAEC), human coronary artery smooth muscle cells (HCASMC), and human umbilical vein endothelial cells (HUVEC) were purchased from commercial suppliers (HCASMSC: Cell Applications, San Diego, CA, USA; HAAF, HAEC, HCAEC, HCASMC: from ScienCell, Carlsbad, CA, USA; HUVEC: PromoCell, Heidelberg, Germany).

**Supplemental Tables**

**Suppl. Table S1: Phenome-wide association study of rs111245230 in UK Biobank.** After correction for multiple testing, associations were considered significant at p<0.0008.

| **Disease group/Disease** | **P** | **OR** | **L95** | **U95** | **N**  **cases** | **N**  **controls** |
| --- | --- | --- | --- | --- | --- | --- |
| ***Cancer*** |  |  |  |  |  |  |
| Bladder cancer | 0.3698 | 0.9146 | 0.7525 | 1.112 | 1,660 | 202,889 |
| Cancer overall | 0,3938 | 1,04 | 0.9498 | 1.14 | 6,951 | 197,598 |
| Cervical cancer | 0.6848 | 0.8957 | 0.5259 | 1.525 | 226 | 204,323 |
| Colorectal cancer | 0.8469 | 1.013 | 0.8847 | 1.161 | 3,138 | 201,411 |
| Female breast cancer | 0.4453 | 1.033 | 0.9498 | 1.124 | 8,207 | 196,342 |
| Kidney cancer | 0.3319 | 0.8908 | 0.7052 | 1.125 | 1,184 | 203,365 |
| Leukaemia | 0.01006 | 1.331 | 1.07 | 1.655 | 939 | 203,610 |
| Lung cancer | 0.222 | 0.8765 | 0.7093 | 1.083 | 1,466 | 203,083 |
| Lymphomas and multiple myeloma | 0.7822 | 1.027 | 0.8493 | 1.242 | 1,570 | 202,979 |
| Ovary cancer | 0.8644 | 1.021 | 0.8014 | 1.302 | 967 | 203,582 |
| Prostate cancer | 0.2833 | 1.053 | 0.9582 | 1.157 | 6,417 | 198,132 |
| Skin cancer_(including melanoma) | 0.9182 | 0.9961 | 0.924 | 1.074 | 10,836 | 193,713 |
| Uterus cancer | 0.04906 | 0.7778 | 0.6052 | 0.9996 | 1,165 | 203,384 |
| ***Cardiovascular diseases*** |  |  |  |  |  |  |
| Aortic valve stenosis | 0.5934 | 1.046 | 0.8858 | 1.236 | 2,015 | 202,534 |
| Atrial fibrillation | 0.7161 | 1.012 | 0.9504 | 1.077 | 15,929 | 188,620 |
| Coronary artery disease | 0.000432 | 1.05941 | 1.01804 | 1.10247 | 38,508 | 406,996 |
| Heart failure | 0.3347 | 1.054 | 0.9474 | 1.172 | 4,987 | 199,562 |
| Hypertension | 0.000247 | 1.066 | 1.03 | 1.103 | 80,443 | 124,106 |
| Peripheral vascular disease | 0.3675 | 1.066 | 0.9274 | 1.226 | 2,840 | 201,709 |
| Stroke | 0.3091 | 1.056 | 0.9507 | 1.173 | 5,101 | 199,448 |
| Venous thromboembolism | 0.6607 | 0.9739 | 0.8654 | 1.096 | 4,330 | 200,219 |
| ***Digestive diseases*** |  |  |  |  |  |  |
| Appendicitis | 0.7824 | 0.9807 | 0.8543 | 1.126 | 3,134 | 201,415 |
| Gallstone | 0.05028 | 0.9255 | 0.8564 | 1 | 10,813 | 193,736 |
| Gastro-oesophageal reflux disease | 0.6749 | 0.9888 | 0.9379 | 1.042 | 23,606 | 180,943 |
| Hiatus hernia (Diaphragmatic hernia) | 0.429 | 0.9804 | 0.9335 | 1.03 | 28,282 | 176,267 |
| Inflammatory bowel disease | 0.1625 | 1.102 | 0.9614 | 1.264 | 2,871 | 201,678 |
| Inguinal hernia | 0.000522 | 1.116 | 1.049 | 1.187 | 14,929 | 189,620 |
| Irritable bowel syndrome | 0.2394 | 1.061 | 0.961 | 1.172 | 5,709 | 198,840 |
| Liver cirrhosis | 0.9338 | 0.9905 | 0.7909 | 1.24 | 1,157 | 203,392 |
| Peptic ulcer | 0.2459 | 1.056 | 0.9631 | 1.158 | 6,689 | 197,860 |
| ***Endocrine disorders*** |  |  |  |  |  |  |
| Diabetes type I | 0.9734 | 1.003 | 0.854 | 1.177 | 2,258 | 202,291 |
| Diabetes type II | 0.9707 | 1.001 | 0.9436 | 1.062 | 18,050 | 186,499 |
| Hyperthyroid | 0.4354 | 0.9293 | 0.773 | 1.117 | 1,837 | 202,712 |
| Hypothyroid | 0.05534 | 0.9374 | 0.8773 | 1.001 | 14,980 | 189,569 |
| ***Eye diseases*** |  |  |  |  |  |  |
| Cataract | 0.581 | 0.9839 | 0.9287 | 1.042 | 19,357 | 185,192 |
| Glaucoma | 0.7412 | 0.9802 | 0.8707 | 1.104 | 4,274 | 200,275 |
| ***Genito-urinary diseases*** |  |  |  |  |  |  |
| Benign prostatic hyperplasia | 0.7907 | 1.01 | 0.9407 | 1.084 | 12,171 | 192,378 |
| Chronic kidney diseases | 0.2874 | 0.9415 | 0.8426 | 1.052 | 5,069 | 199,480 |
| Uterine fibroid | 0.000422 | 0.855 | 0.7836 | 0.9329 | 9,096 | 195,453 |
| ***Musculoskeletal diseases*** |  |  |  |  |  |  |
| Gout | 0.6924 | 1.027 | 0.8995 | 1.173 | 3,254 | 201,295 |
| Hip fracture | 0.1002 | 1.134 | 0.9759 | 1.318 | 2,304 | 202,245 |
| Intervertebral disc disorder | 0.7623 | 1.014 | 0.9256 | 1.111 | 7,061 | 197,488 |
| Osteoarthritis | 0.8926 | 1.003 | 0.9589 | 1.049 | 34,120 | 170,429 |
| Osteoporosis | 0.1279 | 0.9234 | 0.8332 | 1.023 | 6,053 | 198,496 |
| Rheumatoid arthritis | 0.7892 | 1.02 | 0.8842 | 1.176 | 2,835 | 201,714 |
| Sarcoidosis | 0.3927 | 0.8408 | 0.5646 | 1.252 | 429 | 204,120 |
| Sciatica | 0.8915 | 0.9888 | 0.8413 | 1.162 | 2,259 | 202,290 |
| ***Neurological diseases*** |  |  |  |  |  |  |
| Vasculitis | 0.641 | 0.9709 | 0.8576 | 1.099 | 3,925 | 200,624 |
| Alcohol dependency | 0.7878 | 0.9872 | 0.8991 | 1.084 | 6,903 | 197,646 |
| Anxiety | 0.05307 | 0.7313 | 0.5319 | 1.005 | 766 | 203,783 |
| Bipolar | 0.5938 | 0.9443 | 0.7649 | 1.166 | 1,380 | 203,169 |
| Dementia | 0.1819 | 0.9529 | 0.8877 | 1.023 | 12,766 | 191,783 |
| Depression | 0.393 | 1.061 | 0.9258 | 1.217 | 2,976 | 201,573 |
| Epilepsy | 0.137 | 0.8945 | 0.7723 | 1.036 | 3,002 | 201,547 |
| Migraine | 0.1033 | 1.182 | 0.9663 | 1.447 | 1,224 | 203,325 |
| Multiple sclerosis | 0.3973 | 0.9122 | 0.7372 | 1.129 | 1,395 | 203,154 |
| Parkinsons disease | 0.9971 | 0.9995 | 0.7576 | 1.319 | 755 | 203,794 |
| Schizophrenia | 0.641 | 0.9709 | 0.8576 | 1.099 | 3,925 | 200,624 |
| ***Respiratory diseases*** |  |  |  |  |  |  |
| Allergy | 0.5888 | 1.104 | 0.7709 | 1.581 | 409 | 204,140 |
| Asthma | 0.3271 | 1.025 | 0.9754 | 1.078 | 26,134 | 178,415 |
| Chronic obstructive pulmonary disease | 0.4506 | 0.9658 | 0.8823 | 1.057 | 7,555 | 196,994 |
| Hayfever eczema | 0.5275 | 1.038 | 0.925 | 1.164 | 4,316 | 200,233 |
| Lower respiratory infection pneumonia | 0.5577 | 1.019 | 0.9563 | 1.086 | 15,080 | 189,469 |
| Otitis media | 0.3953 | 1.089 | 0.8946 | 1.326 | 1,391 | 203,158 |

**Suppl. Table S2**: **Genes of interest and used Taqman probes.**

| **Gene of interest** | **Taqman probe** |
| --- | --- |
| *CXCL1* | Hs00236937_m1 |
| *CXCL2* | Hs00601975_m1 |
| *CXCL12* | Hs03676656_mH |
| *CX3CR1* | Hs01922583_s1 |
| *CCL2* | Hs00234140_m1 |
| *CCL5* | Hs00982282_m1 |
| *IL1B* | Hs01555410_m1 |
| *IL6* | Hs00174131_m1 |
| *IL10* | Hs00961622_m1 |
| *TGFB1* | Hs00998133_m1 |
| *TNF* | Hs00174128_m1 |
| *ICAM1* | Hs00164932_m1 |
| *ICAM2* | Hs00609563_m1 |
| *SELE* | Hs00174057_m1 |
| *VCAM1* | Hs01003372_m1 |
| *MMP3* | Hs00968305_m1 |
| *MMP9* | Hs00957562_m1 |
| *Svep1* | Mm01346914_m1 |
| *GAPDH* | Hs02758991_g1 |
| *Gapdh* | 4352339E |

**Supplemental Figures**

**
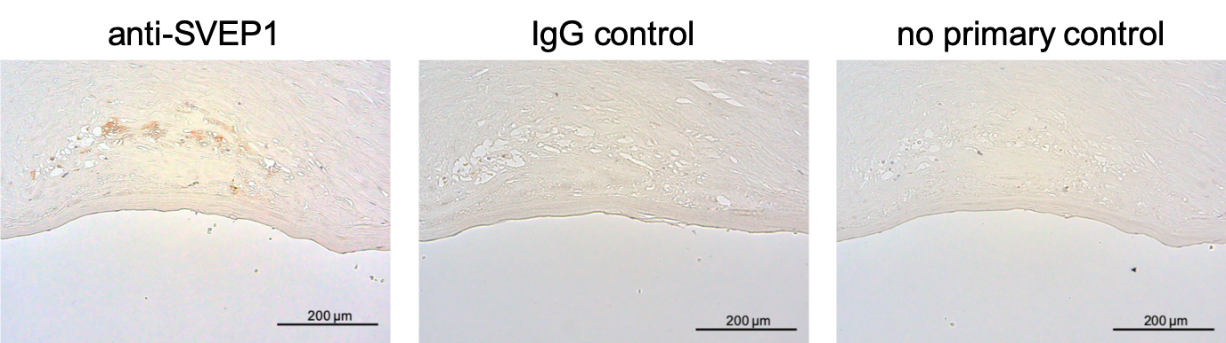
**

**Suppl. Fig. S1: SVEP1 antibody staining and negative controls.** Carotid plaque.


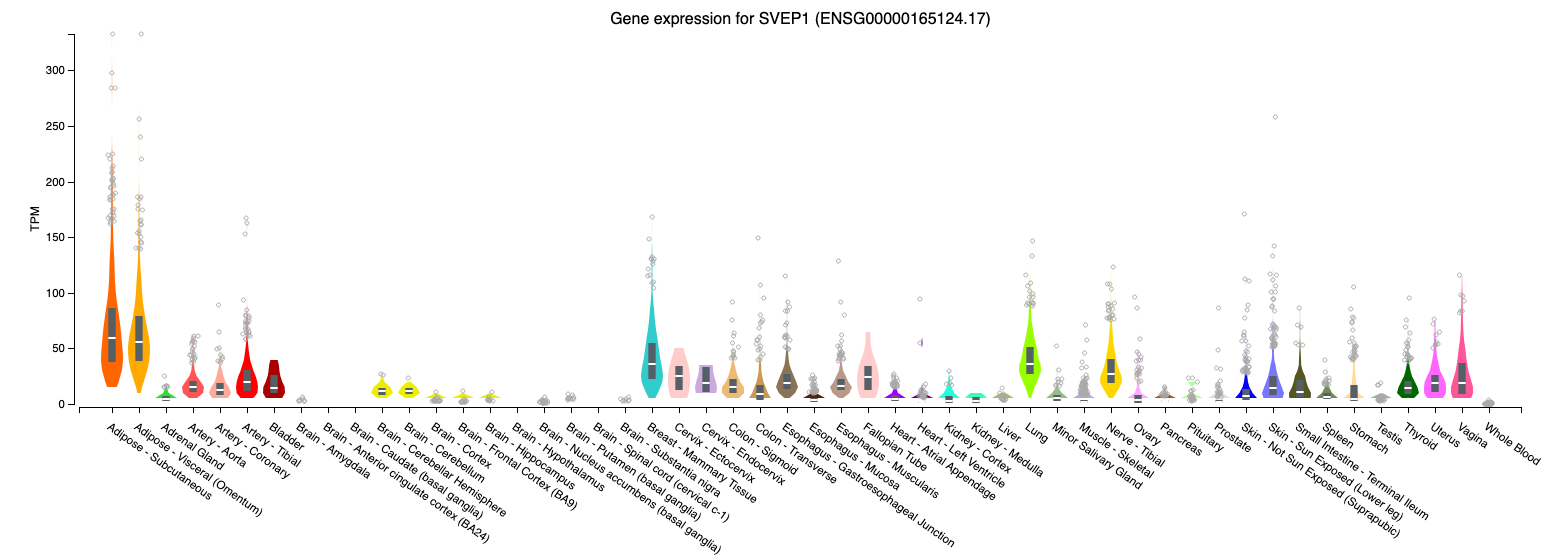


**Suppl. Fig. S2: *SVEP1* expression in different human tissues.** Data source: https://gtexportal.org/home/, accessed on 09-09-2020) [5].

**
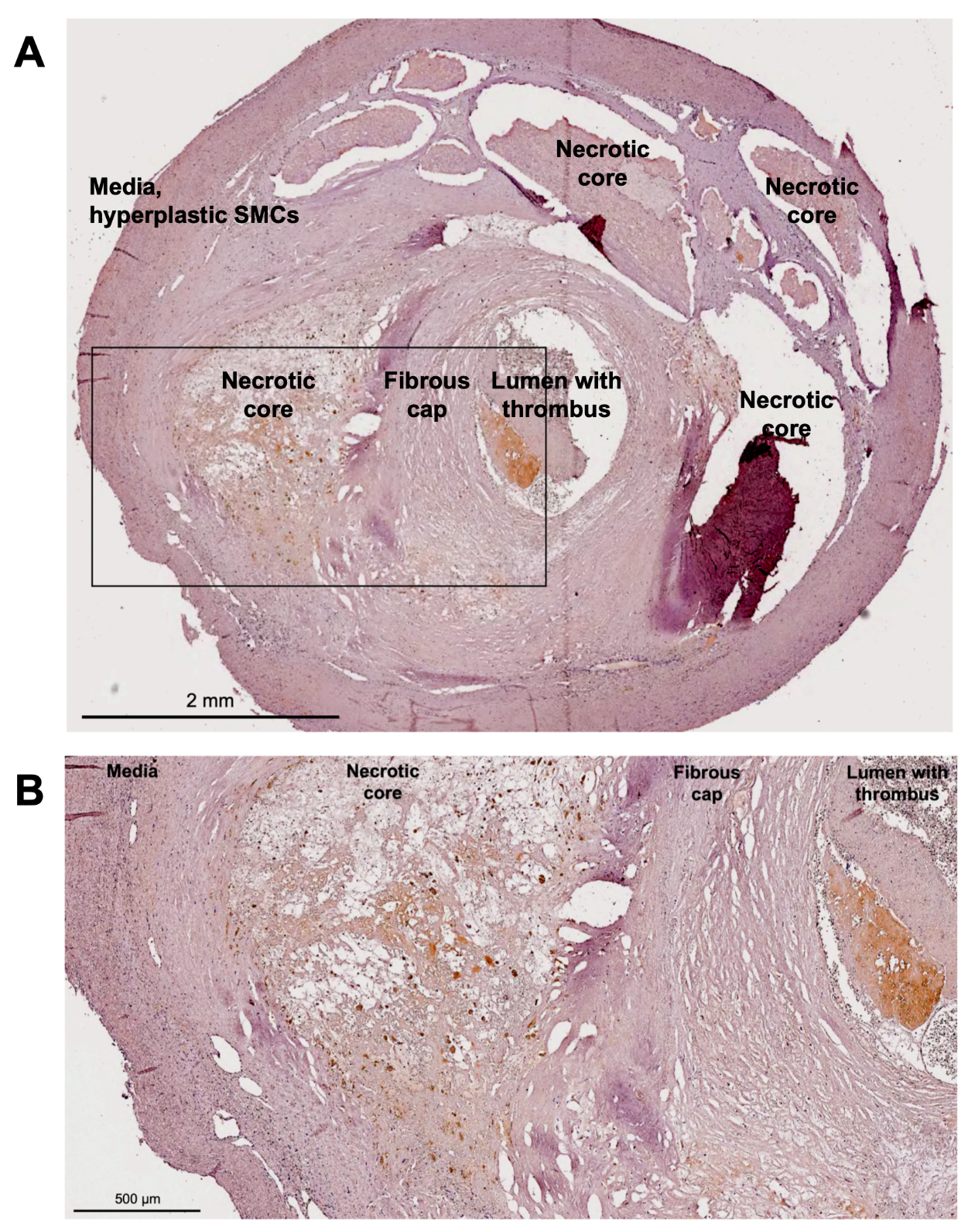
**

**Suppl. Fig. S3: Investigated carotid plaque in Fig. 1C (Individual #1).** **A**. Overview. **B**. Cutting (4x magnification) marked by box in panel A. Anti-SVEP1 staining. *SMCs*, smooth muscle cells.

**
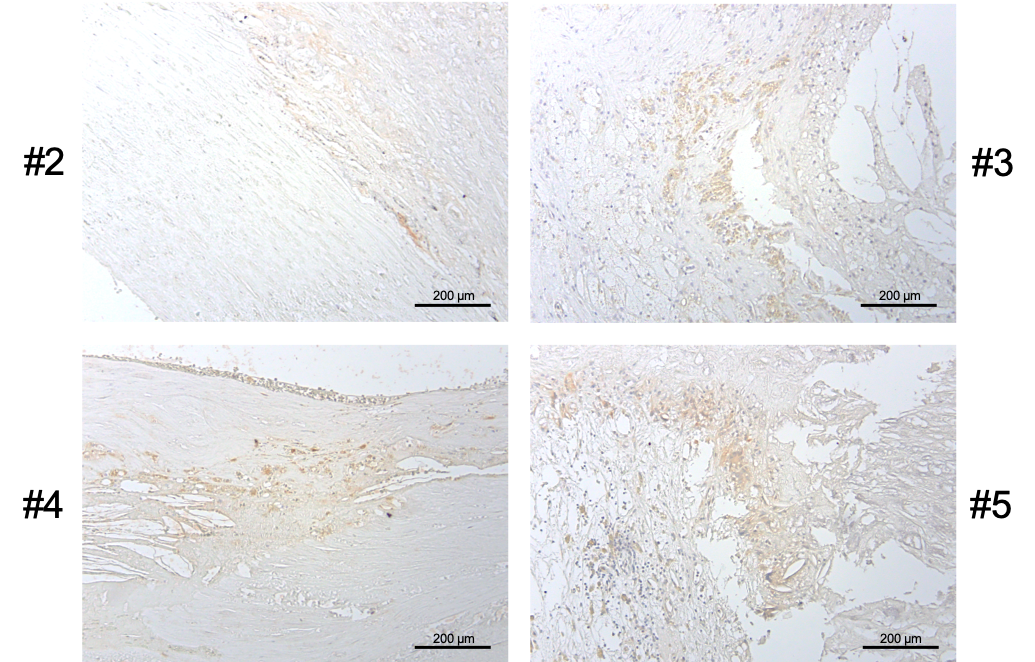
**

**Suppl. Fig. S4: Further carotid plaques staining positive for SVEP1.** Individuals #2-5; Anti-SVEP1 staining.

**
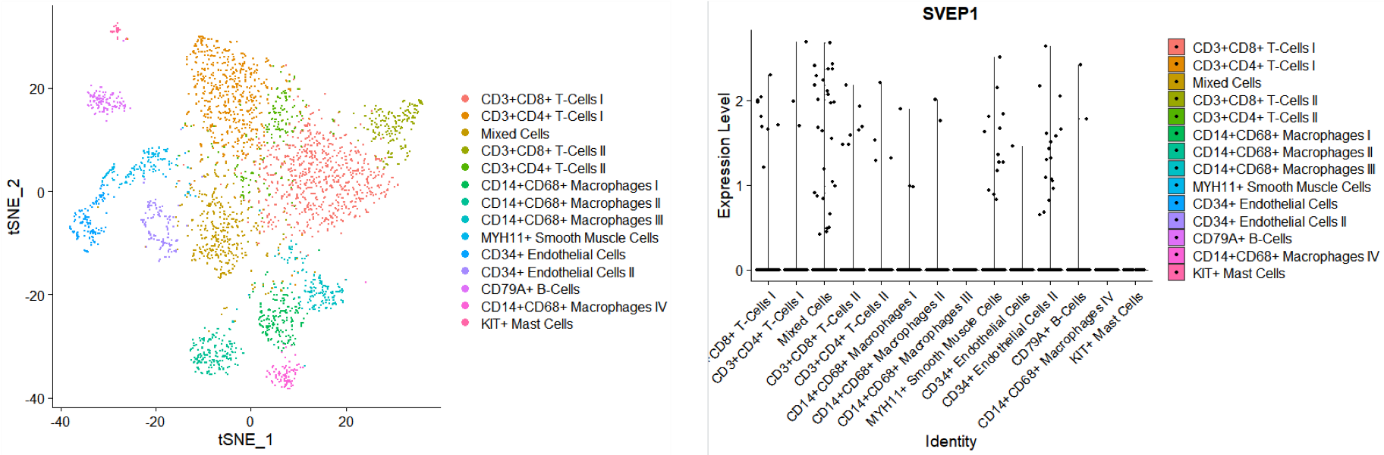
**

**Suppl. Fig. S5: Expression of SVEP1 in carotid artery plaque specimen.** Data from [9].

**Suppl. Fig. S6: Cardiovascular phenotyping of *Svep1*^+/-^ mice by the International Mouse Phenotyping Consortium (IMPC**) [4]**.**

Source: <https://www.mousephenotype.org/data/genes/MGI:1928849>, accessed on 2019-07-31.

**
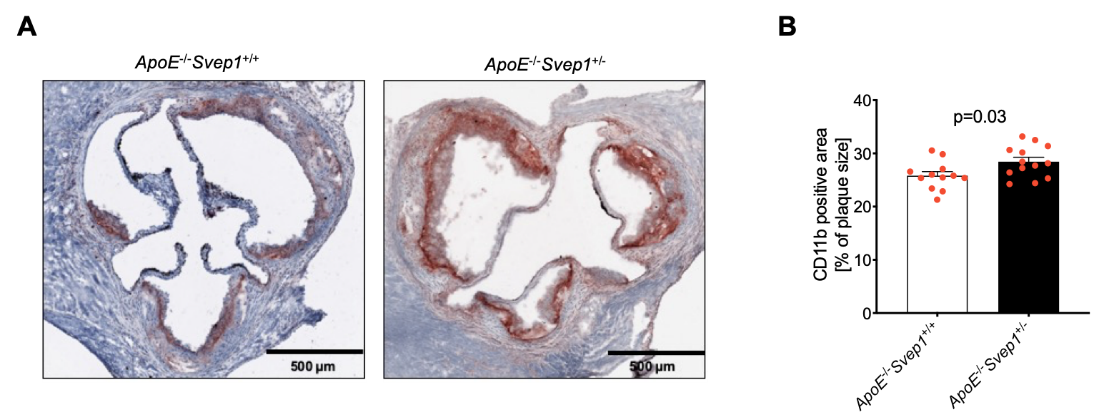
**

**Suppl. Fig. S7: Cd11b positive area in atherosclerotic plaques of *ApoE*^-/-^*Svep1*^+/+^ and *ApoE*^-/-^*Svep1*^+/-^mice. A.** Immunohistochemical staining. **B.** Quantification. Data are mean and s.e.m. Unpaired t-test.

**Suppl. Fig. S8: Quantification of plaque non-classical (Ly6C^low^) monocytes in *ApoE*^-/-^*Svep1*^+/+^ and *ApoE*^-/-^*Svep1*^+/-^ mice on western diet for 12 weeks.** Data are mean and s.e.m. Unpaired t-test.

**Suppl. Fig. S9: Design of adoptive transfer experiment.** *HCD*, western diet.


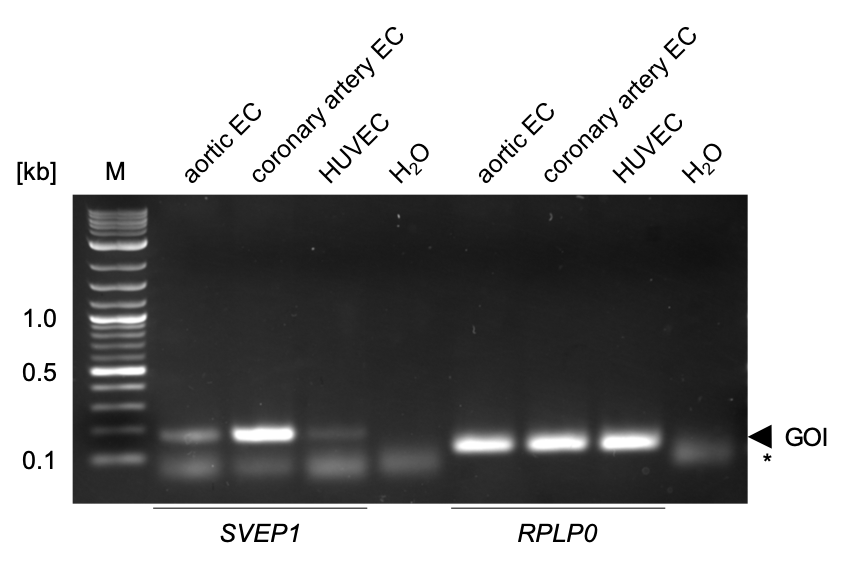


**Suppl. Figure S10**: **Expression of *SVEP1* in different endothelial cells.** GOI depicts the gene of interest, i.e., *SVEP1* (left) or *RPLP0* (right). *, primer dimers. Abbreviations: *EC*, endothelial cell(s); *GOI*, gene of interest; *M*, marker.

**Suppl. Figure S11**: **Expression of *SELE* in HUVEC after incubation with SVEP1 or control.** Data are mean and s.e.m. Paired t-test.

**Supplemental References**

1. Aran D, Looney AP, Liu L, Wu E, Fong V, Hsu A, Chak S, Naikawadi RP, Wolters PJ, Abate AR, Butte AJ, Bhattacharya M (2019) Reference-based analysis of lung single-cell sequencing reveals a transitional profibrotic macrophage. Nat Immunol 20:163–172. doi: 10.1038/s41590-018-0276-y

2. Butler A, Hoffman P, Smibert P, Papalexi E, Satija R (2018) Integrating single-cell transcriptomic data across different conditions, technologies, and species. Nat Biotechnol 36:411–420. doi: 10.1038/nbt.4096

3. Depuydt MA, Prange KH, Slenders L, Örd T, Elbersen D, Boltjes A, de Jager SC, Asselbergs FW, de Borst GJ, Aavik E, Lönnberg T, Lutgens E, Glass CK, Ruijter den HM, Kaikkonen MU, Bot I, Slütter B, van der Laan SW, Ylä-Herttuala S, Mokry M, Kuiper J, de Winther MP, Pasterkamp G (2020) Microanatomy of the Human Atherosclerotic Plaque by Single-Cell Transcriptomics. Circ Res 135:e146–29. doi: 10.1161/CIRCRESAHA.120.316770

4. Dickinson ME, Flenniken AM, Ji X, Teboul L, Wong MD, White JK, Meehan TF, Weninger WJ, Westerberg H, Adissu H, Baker CN, Bower L, Brown JM, Caddle LB, Chiani F, Clary D, Cleak J, Daly MJ, Denegre JM, Doe B, Dolan ME, Edie SM, Fuchs H, Gailus-Durner V, Galli A, Gambadoro A, Gallegos J, Guo S, Horner NR, Hsu C-W, Johnson SJ, Kalaga S, Keith LC, Lanoue L, Lawson TN, Lek M, Mark M, Marschall S, Mason J, McElwee ML, Newbigging S, Nutter LMJ, Peterson KA, Ramirez-Solis R, Rowland DJ, Ryder E, Samocha KE, Seavitt JR, Selloum M, Szoke-Kovacs Z, Tamura M, Trainor AG, Tudose I, Wakana S, Warren J, Wendling O, West DB, Wong L, Yoshiki A, International Mouse Phenotyping Consortium, Jackson Laboratory, Infrastructure Nationale PHENOMIN, Institut Clinique de la Souris (ICS), Charles River Laboratories, MRC Harwell, Toronto Centre for Phenogenomics, Wellcome Trust Sanger Institute, RIKEN BioResource Center, MacArthur DG, Tocchini-Valentini GP, Gao X, Flicek P, Bradley A, Skarnes WC, Justice MJ, Parkinson HE, Moore M, Wells S, Braun RE, Svenson KL, de Angelis MH, Herault Y, Mohun T, Mallon A-M, Henkelman RM, Brown SDM, Adams DJ, Lloyd KCK, McKerlie C, Beaudet AL, Bucan M, Murray SA (2016) High-throughput discovery of novel developmental phenotypes. Nature 537:508–514. doi: 10.1038/nature19356

5. GTEx Consortium (2013) The Genotype-Tissue Expression (GTEx) project. Nat Genet 45:580–585. doi: 10.1038/ng.2653

6. Hashimshony T, Senderovich N, Avital G, Klochendler A, de Leeuw Y, Anavy L, Gennert D, Li S, Livak KJ, Rozenblatt-Rosen O, Dor Y, Regev A, Yanai I (2016) CEL-Seq2: sensitive highly-multiplexed single-cell RNA-Seq. Genome Biol 17:77. doi: 10.1186/s13059-016-0938-8

7. Martens JHA, Stunnenberg HG (2013) BLUEPRINT: mapping human blood cell epigenomes. Haematologica 98:1487–1489. doi: 10.3324/haematol.2013.094243

8. Muraro MJ, Dharmadhikari G, Grün D, Groen N, Dielen T, Jansen E, van Gurp L, Engelse MA, Carlotti F, de Koning EJP, van Oudenaarden A (2016) A Single-Cell Transcriptome Atlas of the Human Pancreas. Cell Syst 3:385–394.e3. doi: 10.1016/j.cels.2016.09.002

9. Verhoeven BAN, Velema E, Schoneveld AH, de Vries J-PPM, de Bruin P, Seldenrijk CA, de Kleijn DPV, Busser E, van der Graaf Y, Moll F, Pasterkamp G (2004) Athero-express: differential atherosclerotic plaque expression of mRNA and protein in relation to cardiovascular events and patient characteristics. Rationale and design. Eur J Epidemiol 19:1127–1133.
